# Metabolomics and microbiome reveal potential root microbiota affecting the alkaloidal metabolome in *Aconitum vilmorinianum* Kom.

**DOI:** 10.1186/s12866-022-02486-1

**Published:** 2022-03-09

**Authors:** Hongrui Li, Hongdi Shi, Peng Xu, Diqiu Yu

**Affiliations:** 1 CAS Key Laboratory of Tropical Plant Resources and Sustainable Use, Xishuangbanna Tropical Botanical Garden, Chinese Academy of Sciences, Kunming, 650223 China; 2 College of Life Sciences, University of Chinese Academy of Sciences, Beijing, 100049 China; 3 State Key Laboratory for Conservation and Utilization of Bio-Resources in Yunnan, Yunnan University, Kunming, 650091 China

**Keywords:** *Aconitum vilmorinianum* Kom., The alkaloidal metabolome, Root microbiota, Bacterial microbiota, Fungal microbiota

## Abstract

**Background:**

The plant microbiome is vital for plant health, fitness, and productivity. Interestingly, plant metabolites and the plant microbiome can influence each other. The combination of metabolomics and microbiome may reveal the critical links between the plant and its microbiome. It is of great significance to agricultural production and human health, especially for Chinese medicine research. *Aconitum vilmorinianum* Kom. is a herb with alkaloid activities, and its roots are the raw material for some Chinese medicines. Former studies have investigated alkaloidal metabolites and antibacterial activities of endophytes in *A. vilmorinianum* roots. However, there are limited reports on the root microbiota that can influence the alkaloidal metabolome of *A. vilmorinianum*.

**Results:**

This research used ultra performance liquid chromatography-tandem mass spectrometry technology and high-throughput sequencing to examine the alkaloidal metabolome, bacterial microbiota, and fungal microbiota in *A. vilmorinianum* roots at two different sites in China. The results revealed that the samples from the two sites were rich in distinct alkaloidal metabolites and recruited significantly different root microbiota. Based on bioinformatics analysis, we found the potential bacterial and fungal microbiota impacting the alkaloidal metabolome in *A. vilmorinianum*.

**Conclusion:**

Our findings reveal the composition of the alkaloidal metabolome, bacterial root microbiota, and fungal root microbiota in *A. vilmorinianum* roots at two different sites. Potential root microbiota that can influence the alkaloidal metabolome of *A. vilmorinianum* are indicated. This study provides a strategy for the cultivation and research of *A. vilmorinianum* and other Chinese herbs.

**Supplementary Information:**

The online version contains supplementary material available at 10.1186/s12866-022-02486-1.

## Background

In nature, plants interact with microorganisms throughout their growth processes, and plants hardly survive without microorganisms [1, 2]. The plant microbiome is vital to plant health, fitness, and productivity [1, 3–5]. A lot of studies have shown that plants provide nutrients for microorganisms, and some microorganisms supply nutrients, minerals, and vitamins to hosts. In addition, beneficial microorganisms can also enhance host plants’ resistance under abiotic and biotic stresses [6, 7]. These discoveries mean that the growth and survival of plants are the common results of plants and the plant microbiome, and the explanation of the plant phenotype according to its own genomes is not sufficient, so more and more researchers regard the plant as a holobiont of plant and its plant microbiome [7–9].

In recent years, microbiome and metabolomics have attracted much attention. Previous research has indicated that metabolites in plants can shape the plant microbiome [2, 10]. It is well understood because microorganisms live inside and outside host plants, and hosts provide the relevant nutrients for these microorganisms. The growth condition of the plant determines what it can give the microorganism. Amazingly, the plant microbiome also can influence the related metabolome in plants [11–16]. For instance, the phyllosphere microorganisms in tobacco can alter Vitamin B6 converting of tobacco [12]. A *Pseudomonas fluorescens* strain in Arabidopsis (*Arabidopsis thaliana*) has been found that it is able to enhance the host’s resistance to a few bacterial pathogens and induce metabolic changes in the host, including indolyl, D-gluconate, and yet unknown metabolites [13]. Inoculation of some bacteria can alter the metabolism of carbohydrates, arginine, and phytoalexins in Arabidopsis leaves [14]. Additionally, the composition of bacterial microbiota in Arabidopsis roots can be influenced by the triterpene biosynthesis of the host, and several root bacteria are involved in the triterpene transformations [16]. A *Paenibacillus* strain has been found that its nitrogen-fixing effect results in increasing the content of some metabolites in poplar [12, 13, 15].

With technological advancements, combining the plant microbiome and the metabolome leads to a better understanding of the interactions between the plant microbiome and hosts, thus promoting the development of agriculture, pharmaceutical research, and other aspects. Some beneficial microorganisms have been found to produce the metabolites that can promote plants growth and protect plants from biotic and abiotic, such as indole acetic acid, gibberellin, cytokinin, 2,3-butanediol, polyamines, lytic enzymes, and 1-aminocyclopropane-1-carboxylate deaminase [17–22]. Nowadays, agricultural production has faced tremendous pressure resulting from various environmental problems, including the continued global population growth, climatic changes, reduced soil fertility, and increased urbanization. Compared with traditional chemical fertilizers, microbial fertilizers are more friendly to humans and the environment at a low price. Besides, microbial fertilizers have great potential to increase crop yields and enhance crop resistance. So the plant microbiome may be used as a strategy to relieve these stresses. Furthermore, the plant microbiome is of great significance to human health. For example, some secondary metabolites from the plant microbiome, especially endophytic fungi, are potentially valuable for treating several diseases [11, 17, 18, 23]. These microorganisms produce compounds with various bioactivities, including cytotoxic, antioxidant, antitumor, antibacterial, and antifungal. Some microorganisms can induce the synthesis of medicinal ingredients in host plants or directly produce compounds similar to the host’s medicinal ingredients [17, 18, 23–25]. The research on these unique microorganisms is likely to advance pharmaceutical-based research on related diseases. The most famous example is the paclitaxel-producing endophyte. Paclitaxel is a well-known antitumor drug. Numerous endophytes have been identified to produce paclitaxel since Stierle, Strobel, and their team isolated a paclitaxel-producing endophytic fungus (*Taxomycesan dreanae*) from *Taxus brevifolia* [26, 27].

The combination of microbiome and metabolomics also has excellent potential in the development of Traditional Chinese medicine (TCM), which is also of positive significance to human health. TCM is a branch of medicine with a history of thousands of years, and it still plays a vital role in human health. However, the studies on how the plant microbiome influence secondary metabolites of Chinese herbs, which are the raw materials of Chinese medicine, are insufficient [28]. The links between certain metabolites and the plant microbiome can improve our knowledge of TCM to help people cultivate Chinese herbs better and promote the application of TCM.

*Aconitum vilmorinianum* Kom., belonging to the family Ranunculaceae, is a perennial crop. The central medicinal part of *A. vilmorinianum* is its roots. The tuberous mother roots (axial roots) and the tuberous son roots (lateral roots) of *A. vilmorinianum* are usually made into Huangcaowu and Fuzi, respectively, which are famous Chinese medicine in southwestern China [29]. *A. vilmorinianum* is also the primary raw material of other well-known Chinese medicines, such as Yunnan Baiyao and Bulleyaconitine A Tablets [30]. People in southwestern China believe that *A. vilmorinianum* possesses healthcare functions, so they use *A. vilmorinianum* roots in dietary therapies [31]. But it is important noted that *A. vilmorinianum* is poisonous when using it without sufficient experience and proper methods. Moreover, the flower of *A. vilmorinianum*, a rare blue-purple one, has a horticultural value in the future [32]. The major bioactive ingredients in *A. vilmorinianum* are alkaloids, particularly diterpenoid alkaloids (such as vilmorrianine A-C and yunaconitine), acting as pain killers and having anti-inflammatory and immunoregulatory roles [30, 33]. The association research on the alkaloidal metabolome and root microbiota in *A. vilmorinianum* will enrich our understanding of the links between specific medicinal ingredients and the root microbiota to identify the members associated with therapeutic ingredients and provide relevant strategies for the cultivation and application of *A. vilmorinianum*. Alkaloidal metabolites and the antimicrobial activities of *A. vilmorinianum* root endophytes have been investigated previously [34, 35]. Still, little is known about which root microbiota can impact the synthesis of alkaloids in *A. vilmorinianum*.

Here, we examined the alkaloidal metabolome and the composition of root microbiota in *A. vilmorinianum* from two different sites in southwestern China, and explored potential root microbiota that can affect the synthesis of alkaloids in *A. vilmorinianum*. Our research has reference value to the cultivation and study of *A. vilmorinianum* and other Chinese herbs.

## Results

### Metabolomic changes in *A. vilmorinianum* roots between Luquan and Weixi

Alkaloids are basic organic compounds containing nitrogen atoms, with antitumor, antiviral, antibacterial, and anti-inflammatory effects [36]. Alkaloids are essential to human health. Lots of plant alkaloids have the potential to treat cancer [24]. The main bioactivity of *A. vilmorinianum* roots is from alkaloidal metabolites. To identify differences in medicinal ingredients between our samples from the two sites in southwestern China, we investigated the alkaloidal metabolome in the samples.

**Fig. 1:**
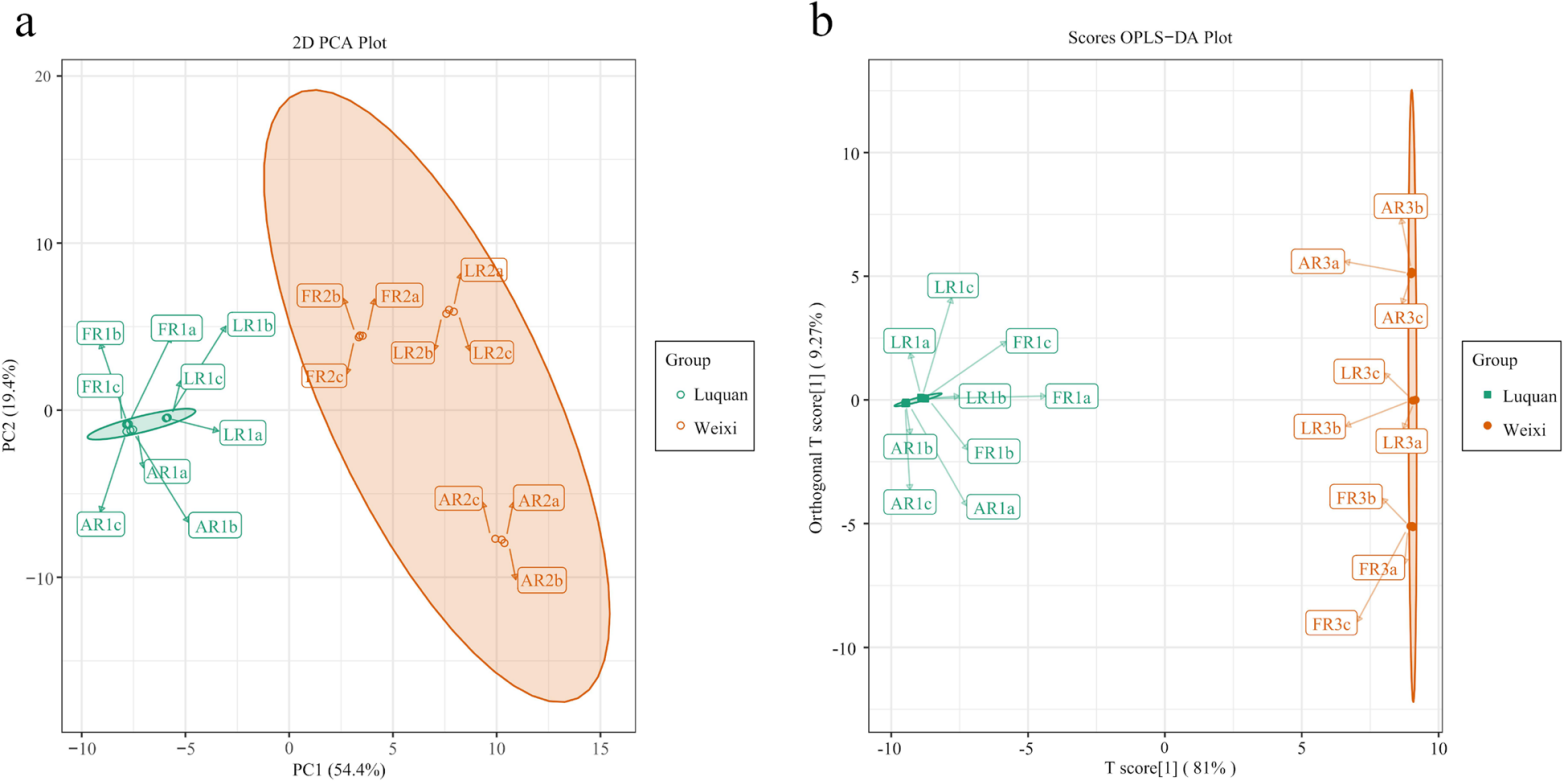
The changes in the alkaloidal metabolome of the samples. **A**, **B** Green represented the samples from Luquan, and orange represented the samples from Weixi. **A** The PCA analysis of the samples from Luquan and Weixi. **B** The OPLS-DA score plot

The principal component analysis (PCA) revealed that the samples from the two sites showed an obvious separation trend (Fig. 1A). To further analyze the differences in the alkaloidal metabolome between the samples and find differential metabolites, we perform orthogonal partial least squares–discriminant analysis (OPLS-DA). The OPLS-DA score also displayed a distinct separation in the samples. The OPLS-DA model exhibited R^2^X = 0.903, R^2^Y = 0.999 and Q^2^ = 0.999, suggesting the model was stable (Fig. 1B and Fig. S1). These results suggested that the differences in the alkaloidal metabolome of the samples between Luquan and Weixi were significant. We selected 75 differential metabolites between the two sites using variable importance in project (VIP) ≥ 1 and a fold change ≥2 or a fold change ≤0.5 as the criteria (Fig. S2). These differential metabolites included vilmorrianine A-C and yunaconitine, which are classically medicinal components in *A. vilmorinianum*. In addition, the samples from Weixi contained aconitine, which has been rarely reported in *A. vilmorinianum* before [32]. The different planting places led to the distinct alkaloidal metabolome in *A. vilmorinianum* roots. However, the specific factors causing these differences need to be further confirmed.

### Diversity, composition, and functional prediction of root microbiota

Plants provide nutrients for the plant microbiome, and their metabolites are the main reason affecting the composition of the plant microbiome [2, 10]. Amusingly, the plant microbiome also impacts the host’s metabolome [11–15]. Studies have indicated that the same medicinal plant has different secondary metabolites at various planting sites, which are likely to be related to the different compositions of microorganisms in different locations [28, 37]. To further examine whether changes in root microbiota followed variations of the alkaloidal metabolome, we performed amplicon sequencing on the root microbiota of *A. vilmorinianum*.

**Fig. 2:**
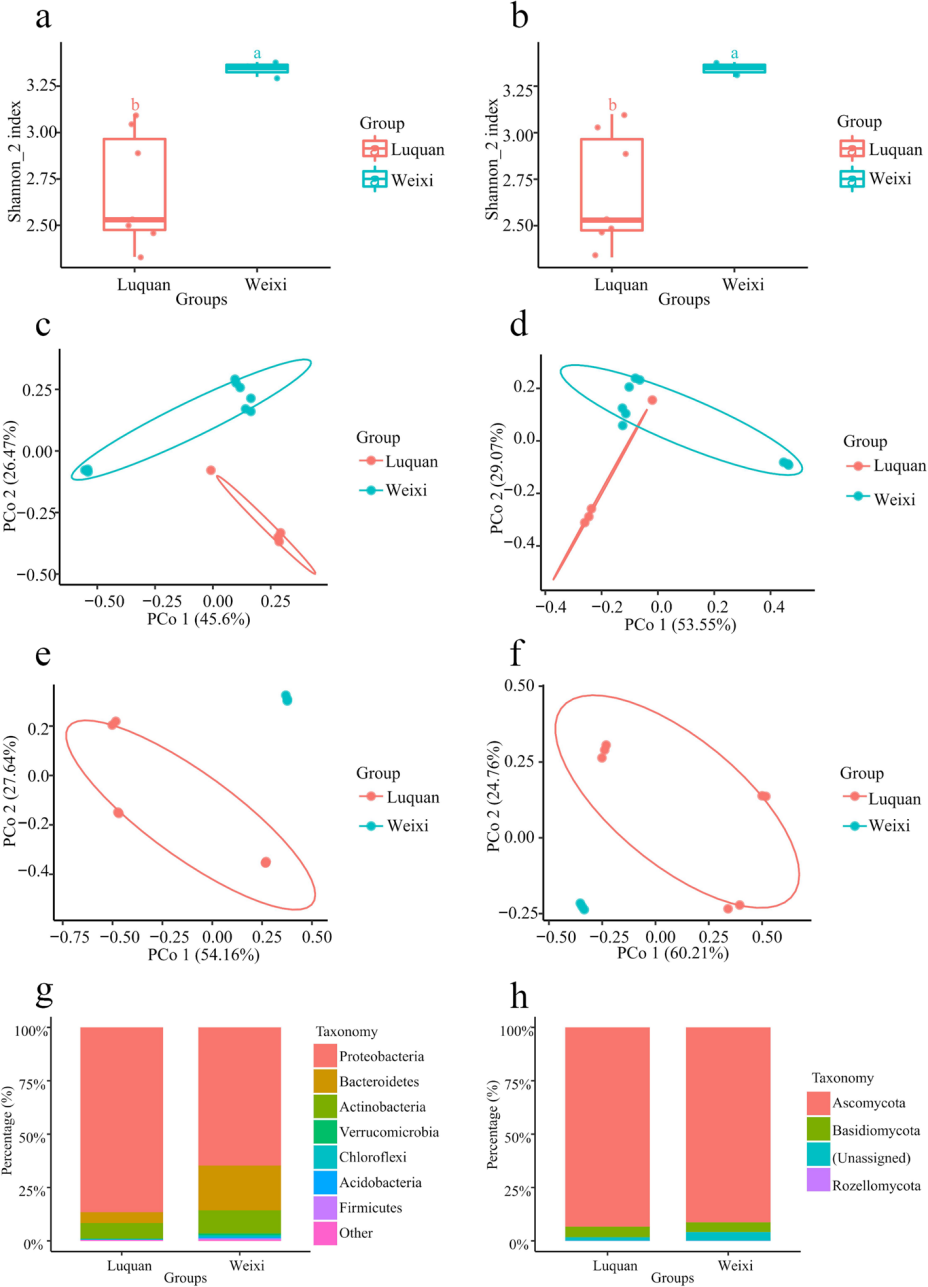
Diversity and composition of root microbiota. **A**, **B** Alpha diversity of root microbiota in the samples (*P* < 0.05, ANOVA, Tukey-HSD test). **A** Shannon index of bacterial microbiota. **B** Shannon index of fungal microbiota. **C**-**F** The principal coordinate analysis (PCoA) based beta diversity of root microbiota in the samples (*P* < 0.05, permutational multivariate analysis of variance (PERMANOVA) by Adonis). **C** PCoA with Bray-Curtis distance of bacterial communities. **D** PCoA with UniFrac distance of bacterial communities. **E** PCoA with Bray-Curtis distance of fungal communities. **F** PCoA with UniFrac distance oft fungal communities. **G**, **H** Phylum-level distribution of root microbiota. **G** The relative abundance of bacterial microbiota. **H** The relative abundance of fungal microbiota

We found significant differences in Shannon indexes of both bacterial and fungal microbiota between Luquan and Weixi (Fig. 2A and B). Shannon index demonstrated that the diversity levels of bacterial and fungal microbiota in Weixi were significantly greater than those in Luquan (Fig. 2A and B), suggesting that *A. vilmorinianum* roots recruited more bacterial and fungal species at Weixi. The principal coordinate analysis (PCoA) of Bray–Curtis and Unifrac distances in bacterial communities exhibited the samples from Luquan and Weixi formed the two distinct clusters (Fig. 2C and D). The PCoA of Bray-Curtis and Unifrac distances in fungal communities also displayed a clear separation in the samples from the two sites (Fig. 2E and F). The PCoA in bacterial and fungal communities indicated that the different planting sites were the central source of the variation in bacterial and fungal microbiota in *A. vilmorinianum* compositions. The relative abundance at the phylum level of the root microbiota revealed that the main bacterial microbiota of the samples were Acidobacteria, Actinobacteria, Bacteroidetes, Chloroflexi, Firmicutes, Proteobacteria, and Verrucomicrobia (Fig. 2G), and the primary root fungal microbiota were Ascomycota and Basidiomycota (Fig. 2H). Besides, the relative abundance of Firmicutes and Proteobacteria were distinct between the two sites, and there was also a significant difference in the relative abundance of unassigned fungi (Figs. S3-S5). These data suggested that *A. vilmorinianum* from Luquan and Weixi recruited distinct root bacterial and fungal microbiota.

**Fig. 3:**
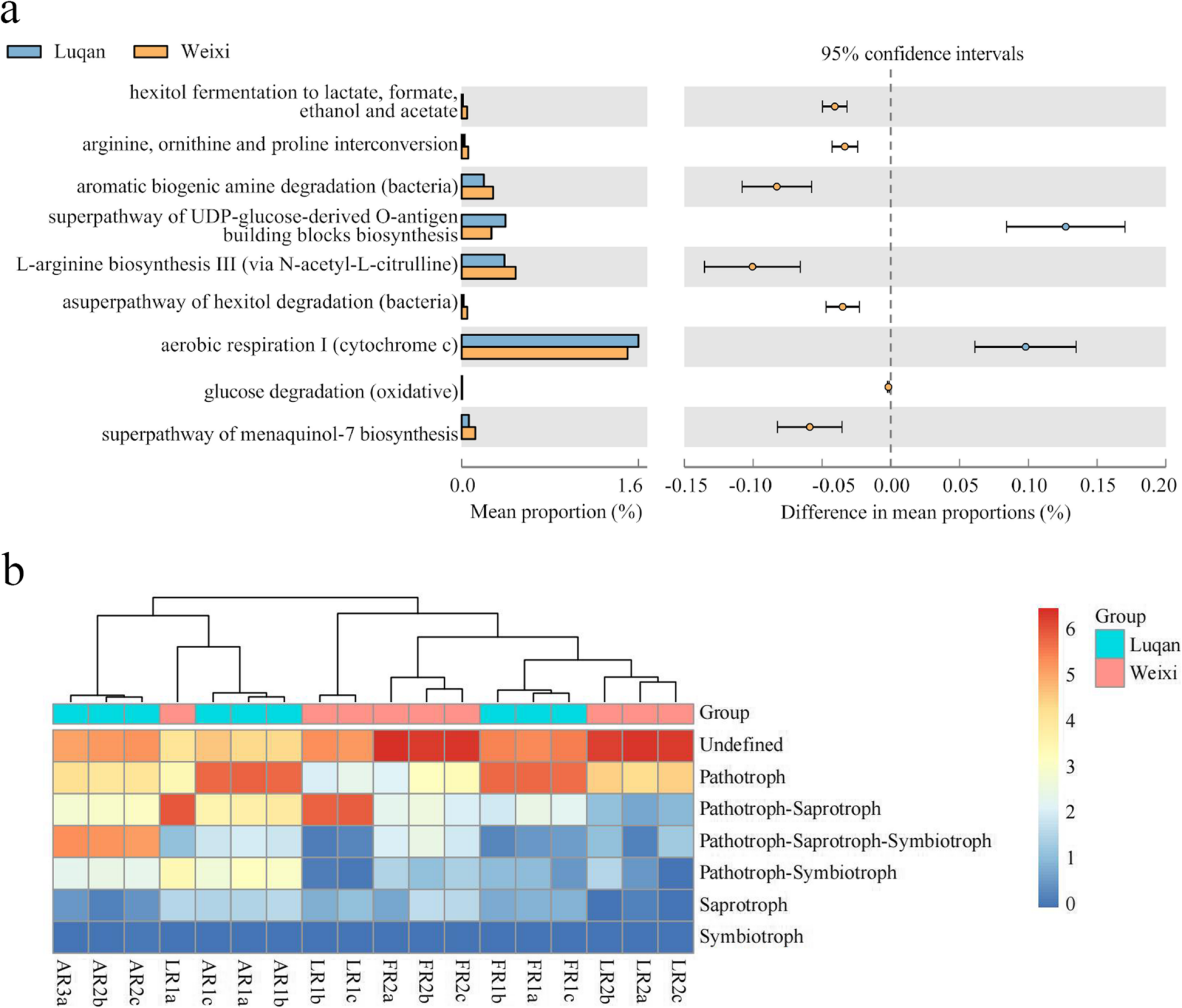
The functional prediction of root microbiota. **A** and **B** Based on results of PICRUSt2 and FUNGuild, respectively. **A** Extended error bar plot of predicted metabolic pathways in bacterial microbiota. There were significant differences in 12 metabolic pathways in the functional abundance of bacterial microbiota from the two sites. Welch’s two-sided t-test and Bonferroni multiple test correction method were performed to test. **B** Heatmap for the composition of fungal functional group (guild) in fungal microbiota. The samples included *A. vilmorinianum* axial roots (AR), lateral roots (LR), and fibrous roots (FR)

To better understand the role of root microbiota recruited by *A. vilmorinianum* at Luquan and Weixi, we predicted the function of bacterial and fungal root microbiota in the samples. According to predictions, there were significantly functional differences in bacterial microbiota between the Luquan and Weixi. The functional abundance of some pathways relating to amino acid metabolism in Weixi’s bacteria was higher than Luquan’s bacteria (Fig. 3A), indicating the distinct bacterial microbiota from two sites were likely to have various contributions to amino acid metabolism. Additionally, the composition of the fungal functional group was also not uniform (Fig. 3B). Therefore, the fungal microbiota recruited by the *A. vilmorinianum* roots from Luquan and Weixi might have different effects on the growth and metabolism of hosts. These predictions suggested *A. vilmorinianum* from the two sites recruited root microbiota, which probably had diverse functions. Nevertheless, more evidence was needed to determine whether these microbial differences led to the host’s alkaloid metabolome differences.

### Potential root microbiota that can affect the alkaloidal synthesis

Given that some studies have found that the plant microbiome can directly or indirectly alter the synthesis of some metabolites in the host plant [11–15], and microbiota analysis suggested that the samples from Luquan and Weixi respectively recruited distinct root microbiota with possibly various functions, especially bacterial root microbiota, which had significant differences in the functional prediction of amino acid synthesis (Fig. 3A). Consequently, these differences in root microbiota were likely to be one of the main reasons for the significantly different alkaloidal metabolome in the samples. We performed microbe-metabolite vectors (MMVEC) analysis and Linear discriminant analysis Effect Size (LEfSe) to explore this possibility further.

**Fig. 4:**
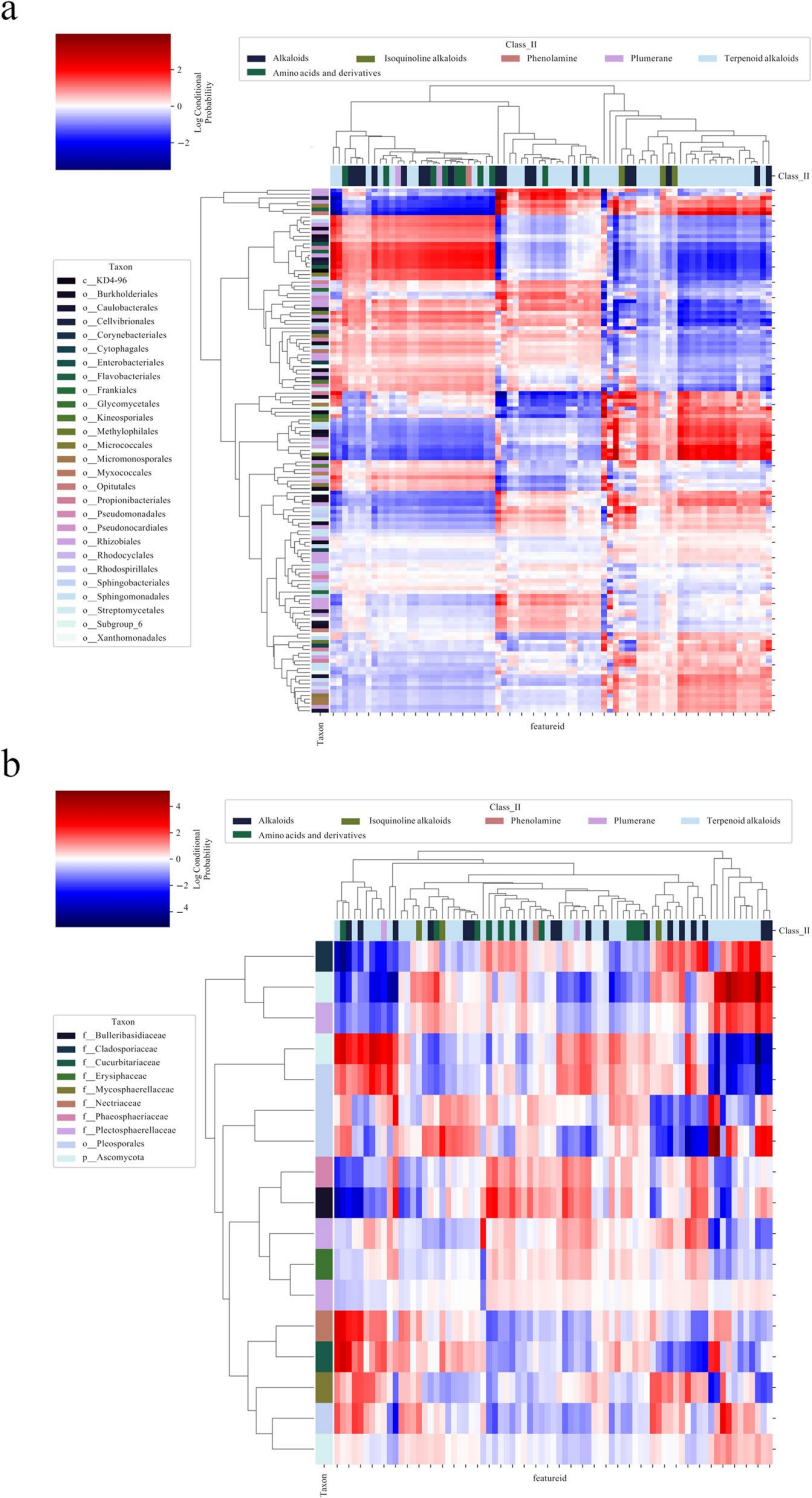
The root microbiota were associated with differential metabolites. **A** and **B** Were cluster heatmaps based on MMVEC analysis, revealing the log conditional probabilities between root microbiota and 75 differential metabolites. The color scale indicated the probabilities of co-occurrence of microorganisms and metabolites. **A** The log conditional probabilities between 137 bacteria and differential metabolites. **B** The log conditional probabilities between 17 fungi and differential metabolites

We determined the conditional probabilities between 75 differential metabolites and parts of the root microbiota, and root microbiota that were not related to differential metabolites were excluded. MMVEC results showed that 137 bacteria and 17 fungi were related to differential metabolites with different conditional probabilities (Fig. 4A and B). We can preliminarily judge the correlation between some microorganisms and alkaloids according to conditional probabilities. The presence of aconitine possibly was related to bacteria *Brevundimonas* and fungus *Cladosporium* (Tables S4, S5, S6 and S7). But it’s important to note that the explanation for this correlation is complex. This correlation between microorganisms and metabolites may be that these microorganisms can influence the synthesis of metabolites, or metabolites can affect the composition of microorganisms.

**Fig. 5:**
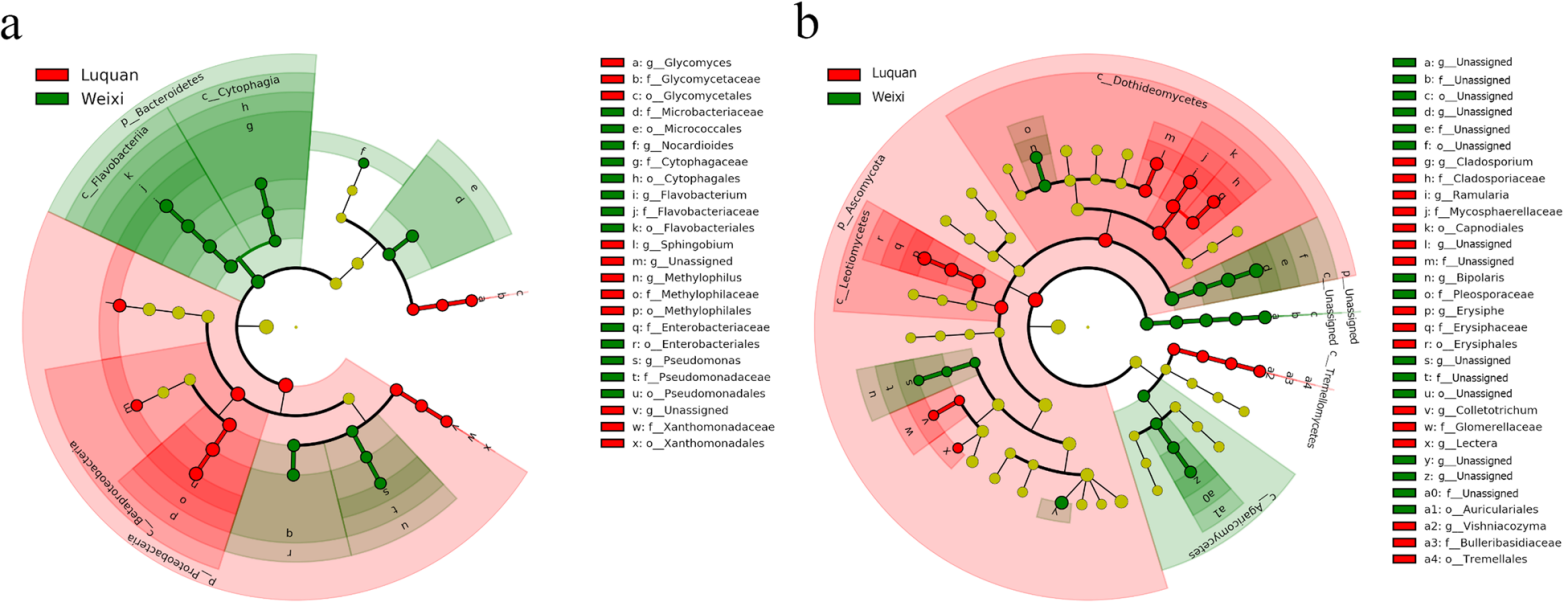
The cladograms of taxa with significant differences between Luquan and Weix. **A** and **B** Represented LEfSe results, which were the taxa with apparent differences between Luquan (green) and Weixi (red). **A** The cladogram of bacterial taxa. **B** The cladogram of fungal taxa

LEfSe results revealed the biomarkers, which were the root microbiota with statistical differences between Luquan and Weixi, and part of fungal biomarkers were not fully annotated. At the family level, bacterial biomarkers in Luquan were Glycomycetaceae, Methylophilaceae, and Xanthomonadaceae, and bacterial biomarkers of the Weixi samples contained Cytophagaceae, Enterobacteriaceae, Flavobacteriaceae, Microbacteriaceae, and Pseudomonadaceae (Fig. 5A and Fig. S6). The Luquan sample’s fungal biomarkers had Bulleribasidiaceae, Cladosporiaceae, Erysiphaceae, Glomerellaceae, and Mycosphaerellaceae, and fungal biomarkers in Weixi were Nectriaceae and Pleosporaceae (Fig. 5B and Fig. S7).

We believed that the root microbiota showing significant differences between the two sites and relating to differential metabolites were more likely to be possible root microbiota we searched. Hence, we selected potential root microbiota that caused the differences in alkaloidal metabolites in *A. vilmorinianum* by combining the results of MMVEC and LEfSe. The potential root bacterial microbiota at the family level were Cytophagaceae, Enterobacteriaceae, Flavobacteriaceae, Glycomycetaceae, Methylophilaceae, Microbacteriaceae, Nocardioidaceae, Pseudomonadacea, Sphingomonadaceae, and Xanthomonadaceae. The potential root fungal microbiota were Bulleribasidiaceae, Cladosporiaceae, Erysiphaceae, Mycosphaerellaceae, Nectriaceae, and Plectosphaerellaceae.

**Fig. 6:**
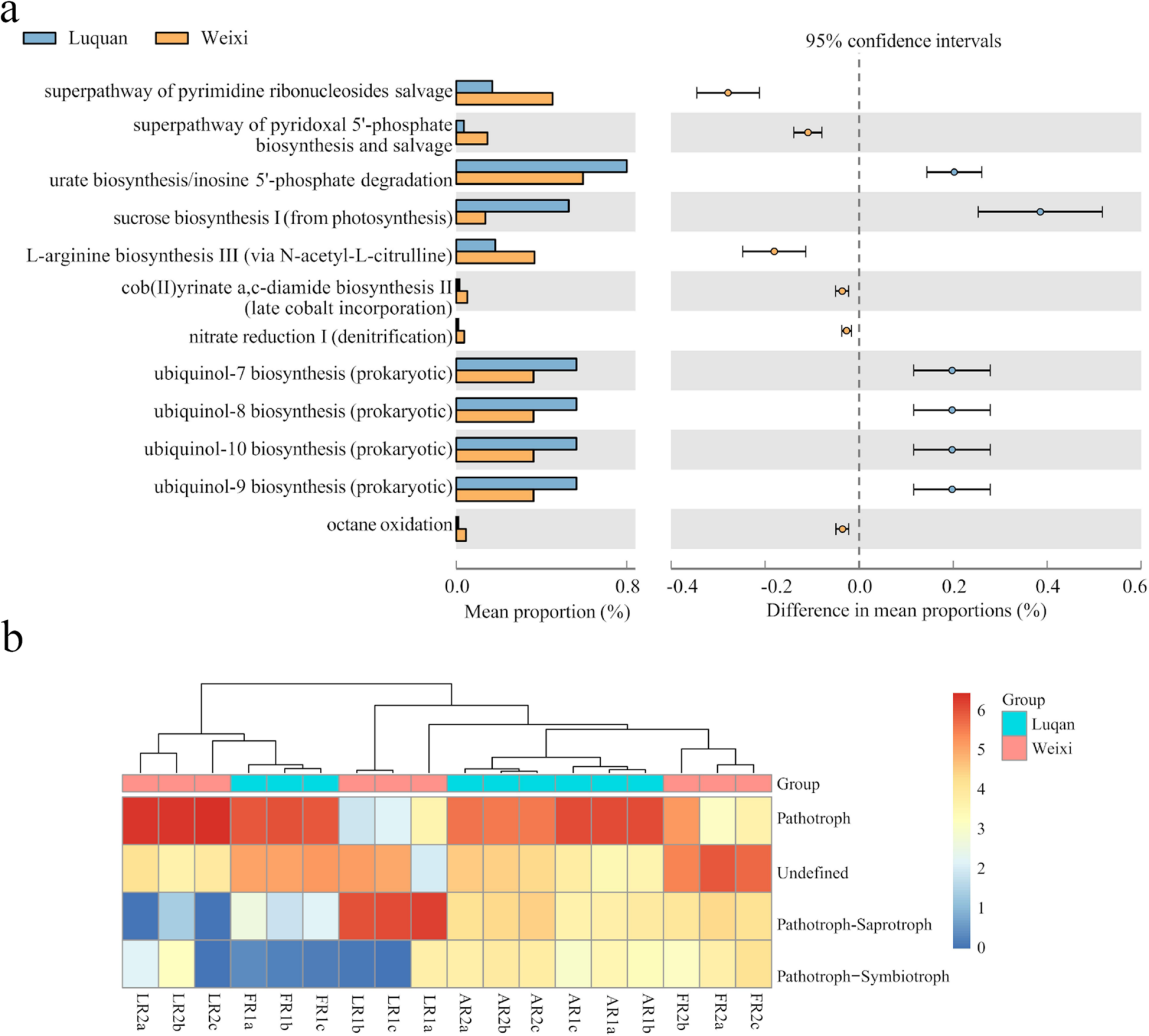
The functional prediction of potential root microbiota that can influence alkaloidal metabolite. **A** and **B** Were inferred from PICRUSt2 and FUNGuild, respectively. **A** Extended error bar plot of predicted metabolic pathways in potential bacteria. In the functional abundance of the samples from Luquan and Weixi, 12 metabolic pathways were significantly different, tested by Welch’s two-sided t-test and Bonferroni multiple test correction method. **B** Heatmap for the composition of fungal functional group (guild) in potential fungi

To better understand how potential root microbiota impact the alkaloidal metabolome of the host, we predicted the functions of potential root microbiota. We found that the target bacteria at the two sites may influence the synthesis of some amino acids (Fig. 6A). These bacteria may cause changes in the alkaloidal metabolome by affecting the host’s amino acid metabolism. The functional prediction of fungi was not as deep as that of bacteria, but we can find that the richness in the guilds was different among the samples, and most of the guilds were Pathotroph and undefined (Fig. 6B). Further experiments are needed to confirm the detailed functions of these potential microorganisms.

## Discussion

The plant is not a completely independent individual in nature, and its growth and development are closely related to the plant microbiome inside and outside the plant [1, 3, 38]. Interestingly, plant metabolites can shape the plant microbiome, and the plant microbiome can also impact the metabolome in hosts [11–16]. The association studies between metabolome and microbiome will provide a deeper understanding of the crucial relationships between specific metabolites and microbiome.

*A. vilmorinianum* is a famous Chinese herb with alkaloidal activity in southwestern China. *A. vilmorinianum* roots are the major raw materials of some Chinese medicines, and *A. vilmorinianum* flowers have potential ornamental value. Previous studies on *A. vilmorinianum* have focused on the alkaloidal composition and endophytic fungi. But so far, little is about the relationship between microorganisms and medicinal ingredients in *A. vilmorinianum*. This research can complement our understanding of this.

### Metabolomic changes in the samples

Existing studies on the metabolome of *A. vilmorinianum* have mainly focused on detecting its alkaloid components, especially diterpene alkaloids. Over 40 diterpenoid alkaloids have been found in *A. vilmorinianum* [32]. The medicinal value of some alkaloids in *A. vilmorinianum* has also been explored. Such as vilmorrianine A has been found the narcotic effects [39]. Studies on whether diverse regions can cause the difference in the medicinal components of *A. vilmorinianum* are limited.

This study used UPLC-MS/MS technology to test the alkaloidal metabolome in *A. vilmorinianum* from two sites. The metabolome analysis indicated that distinct alkaloidal metabolites were enriched in *A. vilmorinianum* roots from Luquan and Weixi (Fig. 1A-B), and 75 differential metabolites were found (Fig. S2). The key ingredient, including yunnaconitine and vilmorrianine A, were up-regulation in the Weixi samples. Amino acids and derivatives were down-regulation in the Luquan samples. Surprisingly, we found that the Weixi’s samples contained aconitine, which had not been formerly reported in *A. vilmorinianum*. Before this, little research has explored the medicinal components of Weixi *A. vilmorinianum*, so aconitine is likely to be unique to the aconite that Weixi grows.

### Microbiota changes in the samples

Among the factors affecting the alkaloidal metabolome of the samples, we investigated *A. vilmorinianum* root microbiota. Former researches have focused on identifying endophytes of *A. vilmorinianum* and their antibacterial activity. Tianpeng et al. found that *Fusarium* and *Penicillium* were the dominant endophytic fungi in *A. vilmorinianum* roots from Kunming, Yunnan Province, China [40]. Zhiying and her group isolated endophytic fungi in *A. vilmorinianum* from Huize County, Yunnan Province, China. Among these fungi, the dominant species in the stems was *Chaetomium*, and the predominant microorganism in roots was *Fusariu* [41]. Zhiying et al. also explored the antibacterial activity of these endophytic fungi [42]. These findings imply that *A. vilmorinianum* is possible to recruit diverse root microbiota in different sites. We performed amplicon sequencing and the related analysis to examine whether changes in the microbiome accompanied changes in the alkaloidal metabolome.

Phylum-level distribution of root microbiota in the samples from Luquan and Weixi displayed that the species compositions of both bacterial and fungal microbiota were the same (Fig. 2G-H), whereas there were significant differences in the alpha diversity, beta diversity, and the relative abundance at the phylum level on some species of bacterial and fungal microbiota in the two group samples (Fig. 2A-F, Figs. S3-S5). Moreover, the functional prediction showed that the functional compositions of bacterial and fungal microbiota from the two sites were various (Fig. 3A-B). Obviously, *A. vilmorinianum* recruited distinct root microbiota at the two environments, and it was seemly that differences in root microbiota contributed to differences in the function of root microbiota.

Because all the sampled *A. vilmorinianum* grew from tuberous roots of last year’s *A. vilmorinianum*, the vertical transmission of the relevant endophytes was probably one of the reasons that caused the distinct root microbiota between the two sites. In addition, the difference in root microbiota was also likely to be related to the different growth environments of the two sites. The temperature may be an essential factor. The annual average temperature of Luquan is 11–24 °C, and the average temperature of Weixi is 7–20 °C. Weixi usually snows in winter, but Luquan rarely snows. Therefore, *A. vilmorinianum* may respond to different temperature conditions by recruiting various root microbiota with different functions.

### Potential root microbiota of affecting alkaloidal synthesis in *A. vilmorinianum*

Our analysis demonstrated that there were significant differences not only in the alkaloid metabolome but also in the composition of root microorganisms between the samples from Luquan and Weixi. Given existing studies, the variation in root microbiota was likely to contribute to the difference in the alkaloid metabolome. Hence, we performed further analysis to explore potential root microbiota that probably altered the host’s alkaloidal metabolome.

We used MMVEC in critical association analysis. MMVEC can predict microbiota–metabolite interactions through neural networking with higher accuracy than traditional methods [43]. MMVEC results showed that 137 bacteria and 17 fungi were associated with 75 differential metabolites (Fig. 4A-B). MMVEC results revealed that In MMVEC results, a fungus *Cladosporium* showed a high conditional probability with aconitine, denoting that the appearance of aconitine in the Weixi sample was possible to be related to this fungus. Kai et al. have found that an endophytic fungus *Cladosporium cladosporioides* in roots of *Aconitum leucostomum*, which belongs to the same genus as *A. vilmorinianum* and can produce aconitine, is able to synthesize aconitine [44]. Therefore, MMVEC results were of a high reference value and can better explain the relationship between differential metabolites and root microbiota. The conditional probability between microorganisms and specific alkaloids can be used as an important basis for related applications.

LEfSe results indicated Luquan samples contained 3 bacterial and 6 fungal biomarkers at the family level, while the Weixi samples contained 5 bacterial and 5 fungal biomarkers at the family level (Fig. 5A-B and Figs. S6-S7). These microorganisms represented by these biomarkers were probably a strategy for *A. vilmorinianum* to survive in different environments. The root microbiota belonging to the biomarkers in Luquan may fit better in the milder climate, and microorganisms on behalf of the Weixi biomarkers may remain active and are helpful for hosts’ survival at a lower temperature. The research and application of microorganisms in different environmental conditions may think about referring to LEfSe results.

The association between microorganisms and metabolites was challenging to explain whether the metabolites impacted the compositions of microorganisms or these microorganisms changed the synthesis of metabolites. The root microbiota, which were associated with related differential metabolites and had significant differences between the two sites, were more likely to potential root microbiota causing metabolomics differences. Thus, through combining the results of MMVEC and LEfSe, we selected10 bacterial and 6 fungal potential microbiota, which probably influenced the alkaloidal metabolome in *A. vilmorinianum*. Except for the discovery of the aconitine-producing fungus *Cladosporium cladosporioides* [44], some microorganisms in the families of potential microbiota also have been identified the functions that affect alkaloid synthesis in plants [18, 45–48]. For instance, Zhilin et al. have found that inoculation with *Pseudomonas* sp. LrLB27 or *Enterobacter* sp. LrBB42 can significantly increase the concentration of several alkaloidal metabolites in *Lycoris radiata* [47]. A *Microbacterium maritypicum* strain from *Ephedra foliate* shows the potential of producing alkaloid and terpenoid compounds [45]. An endophytic fungus *Fusarium solani* from *Camptotheca acuminata* has been found the ability of synthesizing camptothecin and camptothecin analogues [46]. Thus, we believe that these potential microbiota have the value of application and research.

The functional predictions, which were performed using PRICRUSt2 and FUNGuild, showed potential bacteria were likely to be involved in different amino acid metabolism pathways resulting in differences in alkaloid synthesis since alkaloidal synthesis are related to amino acid metabolism (Fig. 6A) and the functions of most fungal potential root microbiota were uncertain (Fig. 6B).

Predictions of potential microbiota functions have changed somewhat from previous functional predictions (Figs. 5A-B and 6A-B). This suggested that there may be other microorganisms that play other important functions, but our results didn’t include these microorganisms. There were similarities in the two functional predictions, implying that these potential microorganisms performed the major relevant functions in the environment. It should be noted that these are only functional predictions and do not fully represent the true function of microorganisms. More experiments are needed to identify and prove the function of microorganisms.

In general, our study can provide a strategy for research and application of *A. vilmorinianum* and other medicinal plants. *A. vilmorinianum* and some Chinese herbs face several problems, including lack of studies on the interactions of secondary metabolites and the plant microbiome [28], the wild resources drastically reducing, and the recent experience of artificial cultivation and introduction is still not sufficient. These problems have put pressure on cultivation and related pharmaceutical research. However, the influence of microorganisms in Chinese herbs on hosts’ secondary metabolites has not been thoroughly studied [28]. Our study is likely to provide strategies for relieving stress. For example, planting *A. vilmorinianum* in the colder environment may consider using the biomarkers in the Weixi samples as the microbial fertilizer (Fig. 5A-B and Figs. S6-S7). To improve the quality of *A. vilmorinianum*, such as raising the content of certain alkaloids, increasing or decreasing the level of certain microorganisms based on MMVEC results in the soil can be taken into account (Fig. 4A-B). Similar studies in other plants can use these microorganisms as the research candidates. In addition, the research on other Chinese herbs can refer to our methods and explore the important microorganisms related to medicinal ingredients.

It must be acknowledged that our results are only based on bioinformatics analyses. Thus, not all potential root microbiota that we have indicated can change alkaloidal synthesis in *A. vilmorinianum*. Further experiments need to isolate and identify these potential root microbiota, and perform relevant functional exploration, such as whether the microorganism affects the host’s alkaloidal metabolome directly or indirectly. However, there are some difficulties that subsequent researches must face. One difficulty that requires the most attention is the long growth cycle and low transplant survival rate of *A. vilmorinianum*. In specific experiments, researchers may consider using the plant material, which is closely related to *A. vilmorinianum*, easy to cultivate, and has a short growth cycle, to replace *A. vilmorinianum*. Although the scope of target microorganisms can be narrowed according to our research, there may still be much work to be done in practice. We recommend focusing on potential root microbiota from families with identified functions. For example, the root bacteria from Enterobacteriaceae, Microbacteriaceae, and Pseudomonadacea, and the root fungi from Cladosporiaceae and Nectriaceae can be given priority.

## Conclusion

The research on combining metabolome and microbiome can help us deepen the understanding of the links between the plant and its microbiome to find the microorganisms that are able to alter the specific metabolite synthesis in the host. We conducted this study to explore the root microbiota that can influence the medicinal components of *A. vilmorinianum*. Metabolome data indicated that there were evident changes in the alkaloidal metabolome of the samples between Luquan and Weixi. Microbiota analysis also showed that *A. vilmorinianum* root recruited distinct bacterial and fungal microbiota, and both bacterial and fungal microbiota probably performed diverse functions in the host’s roots. Through further analysis, we found that 137 bacteria and 17 fungi were associated with differential metabolites, and there were different bacterial and fungal biomarkers in the two samples. Ten bacterial and 6 fungal potential microbiota that caused the differences in the samples were selected. At the family level, the potential bacteria contained Cytophagaceae, Enterobacteriaceae, Flavobacteriaceae, Glycomycetaceae, Methylophilaceae, Microbacteriaceae, Nocardioidaceae, Pseudomonadacea, Sphingomonadaceae, and Xanthomonadaceae, and the potential root fungal microbiota included Bulleribasidiaceae, Cladosporiaceae, Erysiphaceae, Mycosphaerellaceae, Nectriaceae, and Plectosphaerellaceae. The results and methods in this study can provide relevant strategies for the research and application of *A. vilmorinianum* and other medicinal plants. Considering bacteria from Enterobacteriaceae, Microbacteriaceae, and Pseudomonadacea, and fungi from Cladosporiaceae and Nectriaceae, have been found to have the ability to synthesize other alkaloids in other plants, these microorganisms are more likely to influence the alkaloid metabolome in the host plants. Further research and application can focus on these microorganisms.

## Materials and methods

### Sample collection and processing

The samples used in this research were cultivated artificially. In August and October 2020, we collected *A. vilmorinianum* in Luquan County (25.573°N, 102.482°E), Kunming City, and Weixi County (27.338°N, 99.276°E), Diqing Prefecture, respectively, in Yunnan Province, China. These samples reproduced through tuberous roots. We collected *A. vilmorinianum* roots (each sample had three biological replicates), including axial, lateral, and fibrous roots. All the samples collected were healthy plants. After that, we transported these samples to the laboratory on dry ice. After removing soil [49], the samples were frozen in liquid nitrogen. The frozen samples were then stored in a − 80 °C refrigerator. The formal identification of plant material was performed by Professor Dake Zhao of Yunnan University. A voucher specimen of this material with a deposition number LQ15677 has been deposited in the Herbarium of Yunnan University.

### Metabolite extraction and analysis

The samples were ground to powder after freeze-drying, and the 100 mg powder per sample was dissolved in 70% methanol extract [50]. The mixtures were mixed well and placed overnight in a 4 °C refrigerator. After centrifugation, the supernatant of each sample was filtered through a microfiltration membrane [50]. Then the filtered samples were investigated ultra performance liquid chromatography–tandem mass spectrometry (UPLC-MS/MS) technology. Amino acids and their derivatives contain nitrogen and are precursors of alkaloid synthesis. Therefore, amino acids and their derivatives were also considered.

The alkaloids in the samples were determined by the relatively quantitative method. The data of mass spectrum was analyzing by Analyst (v1.6.3, (https://sciex.com/products/software/analyst-software). Total Ions Current (TIC) graphs of each sample were displayed in Figs. S8-S43. Based on the self-build metabolome database MWDB (Metware Biotechnology Co., Ltd. Wuhan, China), the metabolites of the samples were analyzed qualitatively and quantitatively. Qualitative and quantitative analysis results of each sample were showed in Table S1. The PCA was carried out using statistics function prcomp within R (www.r-project.org). Besides, the hierarchical clustering analysis and OPLS-DA were performed using pheatmap and MetaboAnalystR (https://github.com/xia-lab/MetaboAnalystR) packages, respectively, in R [51]. Then, the permutation test (200 permutations) was performed to avoid overfitting. The differential metabolites were selected using VIP ≥ 1 and a fold change ≥2 or a fold change ≤0.5 as the criteria (Tables S2 and S3). The hierarchical clustering analysis of differential metabolites was processed using the R package pheatmap.

### DNA extraction, PCR amplification, and sequencing

The sample DNAs were extracted by the Cetyltrimethylammonium bromide method. An appropriate amount of sterile water was added to dilute the DNAs to 1 ng/ul. The V4-V5 region of the bacterial 16S ribosomal RNA (rRNA) gene and the fungal the internal transcribed spacer 2 (ITS2) region were amplified by Polymerase chain reaction (PCR). We used primers 515F [52] and 907R [53] for bacterial amplification, and primers ITS3 and ITS4 [54] were used for fungal amplification. Each primer contained the barcode as a marker to distinguish the samples. The diluted DNA, primers, Phusion® High-Fidelity PCR Master Mix with GC Buffer (New England Biolabs), and high-fidelity enzymes were used in PCR. The products were mixed in equal amounts based on their concentrations, and then mixed the samples were detected using 2% agarose gel electrophoresis. The Gel Extraction Kit (Qiagen) was used to extract the target bands. A Truseq® DNA PCR-Free Sample Preparation Kit was used for library construction. The qualified libraries were sequenced using Illumina NovaseQ6000. The raw sequence data has been deposited in the Genome Sequence Archive [55] in National Genomics Data Center [56], under accession numbers CRA005007 and CRA005008, which are available at https://ngdc.cncb.ac.cn/gsa.

### Microbiota analysis

The raw sequencing results were separated based on the barcodes and primers. After barcode and primer removal, we used FLASH (v.1.2.7, http://www.cbcb.umd.edu/software/flash) to splice sequences [57]. Quality filtering and dereplication were performed using VSEARCH (v.2.17.0, https://github.com/torognes/vsearch) [58]. USEARCH (v.10.0, https://www.drive5.com/usearch) was used for operational taxonomic unit (OTU) clustering [59]. Subsampling to the same depth was performed using the amplicon package in R [49]. The taxonomy of the OTUs was created using VSEARCH. The SILVA and Unite databases were used to determine the taxonomy of bacterial and fungal OTUs, respectively [60, 61]. Non-bacterial or non-fungal sequences were removed using the amplicon package within R [49]. Alpha diversity, beta diversity, and relative abundance were calculated using USEARCH. Data visualization was carried out using amplicon package in R [49]. To predict the functions of bacterial and fungal microbiota, we performed PICRUSt2 (v.2.4.1, https://github.com/picrust/picrust2) and FUNGuild (https://github.com/UMNFuN/FUNGuild) [62, 63]. Plotting the results of functional prediction used STAMP (v.2.1.3, http://kiwi.cs.dal.ca/Software/STAMP) [64] and the R package pheatmap.

### Exploration of potential root microbiota causing differences of the alkaloidal metabolome

We used differential metabolites and microbiota data to perform MMVEC (v.2.4.1, https://github.com/biocore/mmvec) in QIIME2 [43, 65], and MMVEC results were also visualized using QIIME2-MMVEC. LEfSe was performed using Galaxy LEfSe (https://huttenhower.sph.harvard.edu/galaxy) [66]. The linear discriminant analysis (LDA) score was > 4.00, and the alpha value for the factorial Kruskal–Wallis test among classes was < 0.05 to identify significant differences in taxa between the two sites. We selected the target microorganisms by combining the results of MMVEC and LEfSe, and the microorganisms lacking a taxonomy at the family level were excluded.

## Supplementary Information

**Additional file 1: Sup Fig. S1.** The OPLS-DA model was verified by the permutation test. **Sup Fig. S2.** The cluster heatmap of differential metabolites. The color scale indicated the abundance of metabolites. **Sup Fig. S3.** The change of the relative abundance in Firmicutes. There was a significant difference in the relative abundance in Firmicutes of the samples between Luquan and Weixi (ANOVA, Tukey-HSD test). **Sup Fig. S4.** The change of the relative abundance in Proteobacteria. There was a significant difference in the relative abundance in Proteobacteria of the samples between Luquan and Weixi (ANOVA, Tukey-HSD test). **Sup Fig. S5.** The change of the relative abundance in unassigned fungi. There was a significant difference in the relative abundance in unassigned fungi of the samples between Luquan and Weixi (ANOVA, Tukey-HSD test). **Sup Fig. S6.** The bacterial taxa with their LDA scores. Based on LEfSe results, the taxa were ranked according to their LDA scores. **Sup Fig. S7.** The fungal taxa with their LDA scores. Based on LEfSe results, the taxa were ranked according to their LDA scores. **Sup Figs. S8-S43.** Total ions current (TIC) graphs of each sample. N stood for negative ion mode, P for positive ion mode.

**Additional file 2: Supplementary Table S4.** The conditional probabilities between bacteria and differential metabolites.

**Additional file 3: Supplementary Table S5.** The taxonomy of bacteria associated with differential metabolites.

**Additional file 4: Supplementary Table S6.** The conditional probabilities between bacteria and differential metabolites.

**Additional file 5: Supplementary Table S7.** The taxonomy of fungi associated with differential metabolites.

**Additional file 6: Supplementary Table S1.** The information on alkaloidal metabolites of the samples.

**Additional file 7: Supplementary Table S2.** The differential metabolites (Luquan vs Weixi).

**Additional file 8: Supplementary Table S3.** The differential metabolites (Weixi vs Luquan).

## Data Availability

The amplicon sequencing data are available in the Genome Sequence Archive [30] in National Genomics Data Center [31] https://ngdc.cncb.ac.cn/gsa with accession numbers CRA005007 and CRA005008. The metabolomics data in this research are attached to Supplementary Materials (Tables S1, S2 and S3 and Figs. S8-S43).

## References

[1] Vandenkoornhuyse P, Quaiser A, Duhamel M, Le Van A, Dufresne A (2015). The importance of the microbiome of the plant holobiont. New Phytol.

[2] Jacoby RP, Chen L, Schwier M, Koprivova A, Kopriva S. Recent advances in the role of plant metabolites in shaping the root microbiome. F1000Res. 2020;9. 10.12688/f1000research.21796.1. 10.12688/f1000research.21796.1 PMC7047909 32148778

[3] Turner TR, James EK, Poole PS (2013). The plant microbiome. Genome Biol.

[4] Lareen A, Burton F, Schafer P (2016). Plant root-microbe communication in shaping root microbiomes. Plant Mol Biol.

[5] Yu K, Pieterse CMJ, Bakker PAHM, Berendsen RL (2019). Beneficial microbes going underground of root immunity. Plant Cell Environ.

[6] Berg G, Kusstatscher P, Abdelfattah A, Cernava T, Smalla K (2021). Microbiome modulation-toward a better understanding of plant microbiome response to microbial inoculants. Front Microbiol.

[7] Dastogeer KMG, Tumpa FH, Sultana A, Akter MA, Chakraborty A (2020). Plant microbiome-an account of the factors that shape community composition and diversity. Curr Plant Biol.

[8] Saad MM, Eida AA, Hirt H (2020). Tailoring plant-associated microbial inoculants in agriculture: a roadmap for successful application. J Exp Bot.

[9] Zilber-Rosenberg I, Rosenberg E (2008). Role of microorganisms in the evolution of animals and plants: the hologenome theory of evolution. FEMS Microbiol Rev.

[10] Jacoby RP, Koprivova A, Kopriva S (2021). Pinpointing secondary metabolites that shape the composition and function of the plant microbiome. J Exp Bot.

[11] Etalo DW, Jeon J-S, Raaijmakers JM (2018). Modulation of plant chemistry by beneficial root microbiota. Nat Prod Rep.

[12] Huang S, Zhang J, Tao Z, Lei L, Yu Y, Huang L (2014). Enzymatic conversion from pyridoxal to pyridoxine caused by microorganisms within tobacco phyllosphere. Plant Physiol Biochem.

[13] van de Mortel JE, de Vos RCH, Dekkers E, Pineda A, Guillod L, Bouwmeester K (2012). Metabolic and transcriptomic changes induced in Arabidopsis by the Rhizobacterium Pseudomonas fluorescens SS101. Plant Physiol.

[14] Ryffel F, Helfrich EJN, Kiefer P, Peyriga L, Portais J-C, Piel J (2016). Metabolic footprint of epiphytic bacteria on *Arabidopsis thaliana* leaves. ISME J.

[15] Scherling C, Ulrich K, Ewald D, Weckwerth W (2009). A metabolic signature of the beneficial interaction of the endophyte *Paenibacillus* sp isolate and in vitro-grown poplar plants revealed by metabolomics. Mol Plant-Microbe Interact.

[16] Huang AC, Jiang T, Liu Y-X, Bai Y-C, Reed J, Qu B (2019). A specialized metabolic network selectively modulates Arabidopsis root microbiota. Science.

[17] Fadiji AE, Babalola OO (2020). Elucidating mechanisms of endophytes used in plant protection and other bioactivities with multifunctional prospects. Front Bioeng Biotechnol.

[18] Hardoim PR, van Overbeek LS, Berg G, Pirttila AM, Compant S, Campisano A (2015). The hidden world within plants: ecological and evolutionary considerations for defining functioning of microbial endophytes. Microbiol Mol Biol Rev.

[19] Johnston-Monje D, Raizada MN (2011). Conservation and diversity of seed associated endophytes in Zea across boundaries of evolution, ethnography and ecology. PLoS One.

[20] Perrig D, Boiero ML, Masciarelli OA, Penna C, Ruiz OA, Cassan FD (2007). Plant-growth-promoting compounds produced by two agronomically important strains of Azospirillum brasilense, and implications for inoculant formulation. Appl Microbiol Biotechnol.

[21] Taghavi S, Garafola C, Monchy S, Newman L, Hoffman A, Weyens N (2009). Genome survey and characterization of endophytic Bacteria exhibiting a beneficial effect on growth and development of poplar trees. Appl Environ Microbiol.

[22] White JF, Kingsley KL, Zhang Q, Verma R, Obi N, Dvinskikh S (2019). Review: endophytic microbes and their potential applications in crop management. Pest Manag Sci.

[23] Kusari S, Hertweck C, Spitellert M (2012). Chemical ecology of endophytic fungi: origins of secondary metabolites. Chem Biol.

[24] Kharwar RN, Mishra A, Gond SK, Stierle A, Stierle D (2011). Anticancer compounds derived from fungal endophytes: their importance and future challenges. Nat Prod Rep.

[25] Rana KL, Kour D, Sheikh I, Dhiman A, Yadav N, Yadav AN, Yadav AN, Mishra S, Singh S, Gupta A (2019). Endophytic fungi: biodiversity, ecological significance, and potential industrial applications. Recent advancement in white biotechnology through fungi, vol 1: diversity and enzymes perspectives.

[26] Stierle A, Strobel G, Stierle D (1993). Taxol and Taxane production by *Taxomyces-andreanae*, an endophytic fungus of Pacific yew. Science.

[27] Li G, Lou H-X (2018). Strategies to diversify natural products for drug discovery. Med Res Rev.

[28] Huang W, Long C, Lam E (2018). Roles of plant-associated microbiota in traditional herbal medicine. Trends Plant Sci.

[29] Li M, He J, Jiang L-L, Ng ES-K, Wang H, Lam FF-Y (2013). The anti-arthritic effects of *Aconitum vilmorinianum*, a folk herbal medicine in southwestern China. J Ethnopharmacol.

[30] Li YG, Mou FJ, Li KZ (2021). De novo RNA sequencing and analysis reveal the putative genes involved in diterpenoid biosynthesis in *Aconitum vilmorinianum* roots. 3 Biotech.

[31] Wang H, Liu B, Zhan R, He F, Wu J, Liu Y (2014). Study on Diterpenoid alkaloids from *Aconitum vilmorinianum* Kom. J Yunnan Agric Univ.

[32] Li X, He J, He S, Meng J (2017). Advances in the study of *Aconitum vilmorinianum* Kom. J West China For Sci.

[33] Ding LS, Chen YZ, Wu FE (1991). Diterpenoid alkaloids from *Aconitum-vilmorrianum*. Planta Med.

[34] Luo P, Shu Y, Zhu L, Ding Z, Cai L (2020). Secondary metabolites of *Fusarium sambucinum* B10.2 from *Aconitum vilmorinianum* Kom. Nat Prod Res Dev.

[35] Li Z, Yang YL, Li S, Chen Y, Wu S (2009). Antibacterial activity of endophytic fungi in *Aconitum vilmorinianum* Kom. Lishizhen Med Materia Med Res.

[36] Casciaro B, Mangiardi L, Cappiello F, Romeo I, Loffredo MR, Iazzetti A (2020). Naturally-occurring alkaloids of plant origin as potential antimicrobials against antibiotic-resistant infections. Molecules.

[37] Koeberl M, Schmidt R, Ramadan EM, Bauer R, Berg G (2013). The microbiome of medicinal plants: diversity and importance for plant growth, quality, and health. Front Microbiol.

[38] Sasse J, Martinoia E, Northen T (2018). Feed your friends: do plant exudates shape the root microbiome?. Trends Plant Sci.

[39] Zhu Y, Zhu R. Research on Chinese *Aconitum* VII. Alkaloids in *Aconitum vilmorinianum* Kom. roots. Acta Pharm Sin. 1965;12(3):167–70.

[40] Yin T, Yu J, Wang J, Cai l, Ding Z. (2016). Isolation and identification of endophytic fungi from *Aconitum vilmorinianum* Kom. roots. China Med Herald.

[41] Li Z, Li S, Yang L, Chen Y, Wu S. Isolation and preliminary identification of endophytic Fungi from *Aconitum vilmorinianum* Kom. in Southern Yunnan. Chin J Ethnomed Ethnopharm. 2008;17(7):10–2.

[42] Li Z, Yang L, Li LS, Chen Y, Wu S. Screening of antimicrobial activity of endophytic Fungi from *Aconitum vilmorinianum* Kom. in Southern Yunnan. Lishizhen Med Materia Med Res. 2009;20(5):1027–9.

[43] Morton JT, Aksenov AA, Nothias LF, Foulds JR, Quinn RA, Badri MH (2019). Learning representations of microbe-metabolite interactions. Nat Methods.

[44] Yang K, Liang J, Li Q, Kong X, Chen R, Jin Y (2013). *Cladosporium* cladosporioides XJ-AC03, an aconitine-producing endophytic fungus isolated from *Aconitum leucostomum*. World J Microbiol Biotechnol.

[45] Ghiasvand M, Makhdoumi A, Matin MM, Vaezi J (2020). Exploring the bioactive compounds from endophytic bacteria of a medicinal plant: *Ephedra foliata* (Ephedrales: Ephedraceae). Orient Pharm Exp Med.

[46] Kusari S, Zuhlke S, Spiteller M (2009). An endophytic fungus from Camptotheca acuminata that produces Camptothecin and analogues. J Nat Prod.

[47] Liu Z, Zhou J, Li Y, Wen J, Wang R (2020). Bacterial endophytes from Lycoris radiata promote the accumulation of Amaryllidaceae alkaloids. Microbiol Res.

[48] Dowarah B, Agarwal H, Krishnatreya DB, Sharma PL, Kalita N, Agarwala N (2021). Evaluation of seed associated endophytic bacteria from tolerant chilli cv. Firingi Jolokia for their biocontrol potential against bacterial wilt disease. Microbiol Res.

[49] Zhang J, Liu Y-X, Zhang N, Hu B, Jin T, Xu H (2019). NRT1.1B is associated with root microbiota composition and nitrogen use in field-grown rice. Nat Biotechnol.

[50] Li S, Deng B, Tian S, Guo M, Liu H, Zhao X (2021). Metabolic and transcriptomic analyses reveal different metabolite biosynthesis profiles between leaf buds and mature leaves in Ziziphus jujuba mill. Food Chem.

[51] Chong J, Xia J (2018). MetaboAnalystR: an R package for flexible and reproducible analysis of metabolomics data. Bioinformatics.

[52] Caporaso JG, Lauber CL, Walters WA, Berg-Lyons D, Lozupone CA, Turnbaugh PJ (2011). Global patterns of 16S rRNA diversity at a depth of millions of sequences per sample. Proc Natl Acad Sci U S A.

[53] Armitage DW, Gallagher KL, Youngblut ND, Buckley DH, Zinder SH (2012). Milimeter-scale patterns of phylogenetic and trait diversity in a salt marsh microbial mat. Front Microbiol.

[54] Fujita SI, Senda Y, Nakaguchi S, Hashimoto T (2001). Multiplex PCR using internal transcribed spacer 1 and 2 regions for rapid detection and identification of yeast strains. J Clin Microbiol.

[55] Chen T, Chen X, Zhang S, Zhu J, Tang B, Wang A, et al. The genome sequence archive family: toward explosive data growth and diverse data types. Genomics Proteomics Bioinformatics. 2021. 10.1016/j.gpb.2021.08.001. 10.1016/j.gpb.2021.08.001 PMC9039563 34400360

[56] Xue Y, Bao Y, Zhang Z, Zhao W, Xiao J, He S (2021). Database Resources of the National Genomics Data Center, China National Center for Bioinformation in 2021. Nucleic Acids Res.

[57] Magoc T, Salzberg SL (2011). FLASH: fast length adjustment of short reads to improve genome assemblies. Bioinformatics.

[58] Rognes T, Flouri T, Nichols B, Quince C, Mahe F (2016). VSEARCH: a versatile open source tool for metagenomics. Peerj.

[59] Edgar RC (2010). Search and clustering orders of magnitude faster than BLAST. Bioinformatics.

[60] Abarenkov K, Allan Z, Piirmann T, Pöhönen R, Ivanov F, Nilsson RH, Kõljalg U (2021). UNITE USEARCH/UTAX release for Fungi. UNITE Community.

[61] Quast C, Pruesse E, Yilmaz P, Gerken J, Schweer T, Yarza P (2013). The SILVA ribosomal RNA gene database project: improved data processing and web-based tools. Nucleic Acids Res.

[62] Nguyen NH, Song Z, Bates ST, Branco S, Tedersoo L, Menke J (2016). FUNGuild: an open annotation tool for parsing fungal community datasets by ecological guild. Fungal Ecol.

[63] Douglas GM, Maffei VJ, Zaneveld JR, Yurgel SN, Brown JR, Taylor CM (2020). PICRUSt2 for prediction of metagenome functions. Nat Biotechnol.

[64] Parks DH, Tyson GW, Hugenholtz P, Beiko RG (2014). STAMP: statistical analysis of taxonomic and functional profiles. Bioinformatics.

[65] Bolyen E, Rideout JR, Dillon MR, Bokulich N, Abnet CC, Al-Ghalith GA (2019). Reproducible, interactive, scalable and extensible microbiome data science using QIIME 2. Nat Biotechnol.

[66] Segata N, Izard J, Waldron L, Gevers D, Miropolsky L, Garrett WS (2011). Metagenomic biomarker discovery and explanation. Genome Biol.

