# Supplementary material for: Metabolomics and microbiome reveal potential root microbiota affecting the alkaloidal metabolome in Aconitum vilmorinianum Kom
Source: BMC Microbiol. 2022 Mar 9;22:70. doi: 10.1186/s12866-022-02486-1 (PMC8905797; doi:10.1186/s12866-022-02486-1)
Supplement: Supplementary file 1 — Additional file 1: Sup Fig. S1. The OPLS-DA model was verified by the permutation test. Sup Fig. S2. The cluster heatmap of differential metabolites. The color scale indicated the abundance of metabolites. Sup Fig. S3. The change of the relative abundance in Firmicutes. There was a significant difference in the relative abundance in Firmicutes of the samples between Luquan and Weixi (ANOVA, Tukey-HSD test). Sup Fig. S4. The change of the relative abundance in Proteobacteria. There was a significant difference in the relative abundance in Proteobacteria of the samples between Luquan and Weixi (ANOVA, Tukey-HSD test). Sup Fig. S5. The change of the relative abundance in unassigned fungi. There was a significant difference in the relative abundance in unassigned fungi of the samples between Luquan and Weixi (ANOVA, Tukey-HSD test). Sup Fig. S6. The bacterial taxa with their LDA scores. Based on LEfSe results, the taxa were ranked according to their LDA scores. Sup Fig. S7. The fungal taxa with their LDA scores. Based on LEfSe results, the taxa were ranked according to their LDA scores. Sup Figs. S8-S43. Total ions current (TIC) graphs of each sample. N stood for negative ion mode, P for positive ion mode. [file 12866_2022_2486_MOESM1_ESM.docx]

**Supplementary Figures**


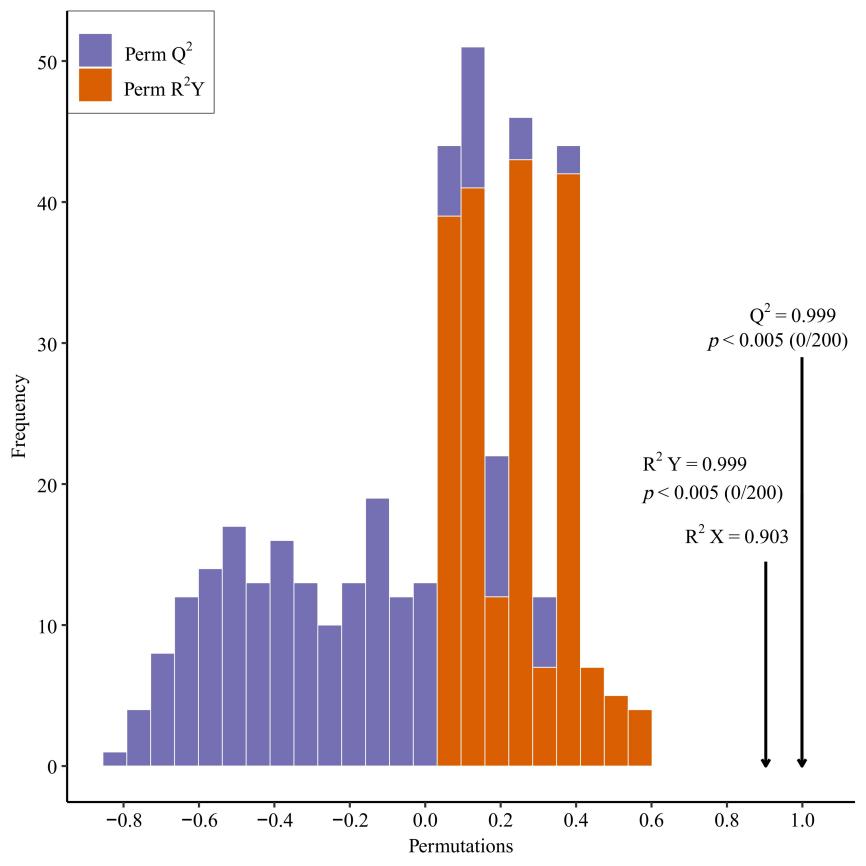


**Sup Fig. S1** The OPLS-DA model was verified by the permutation test.


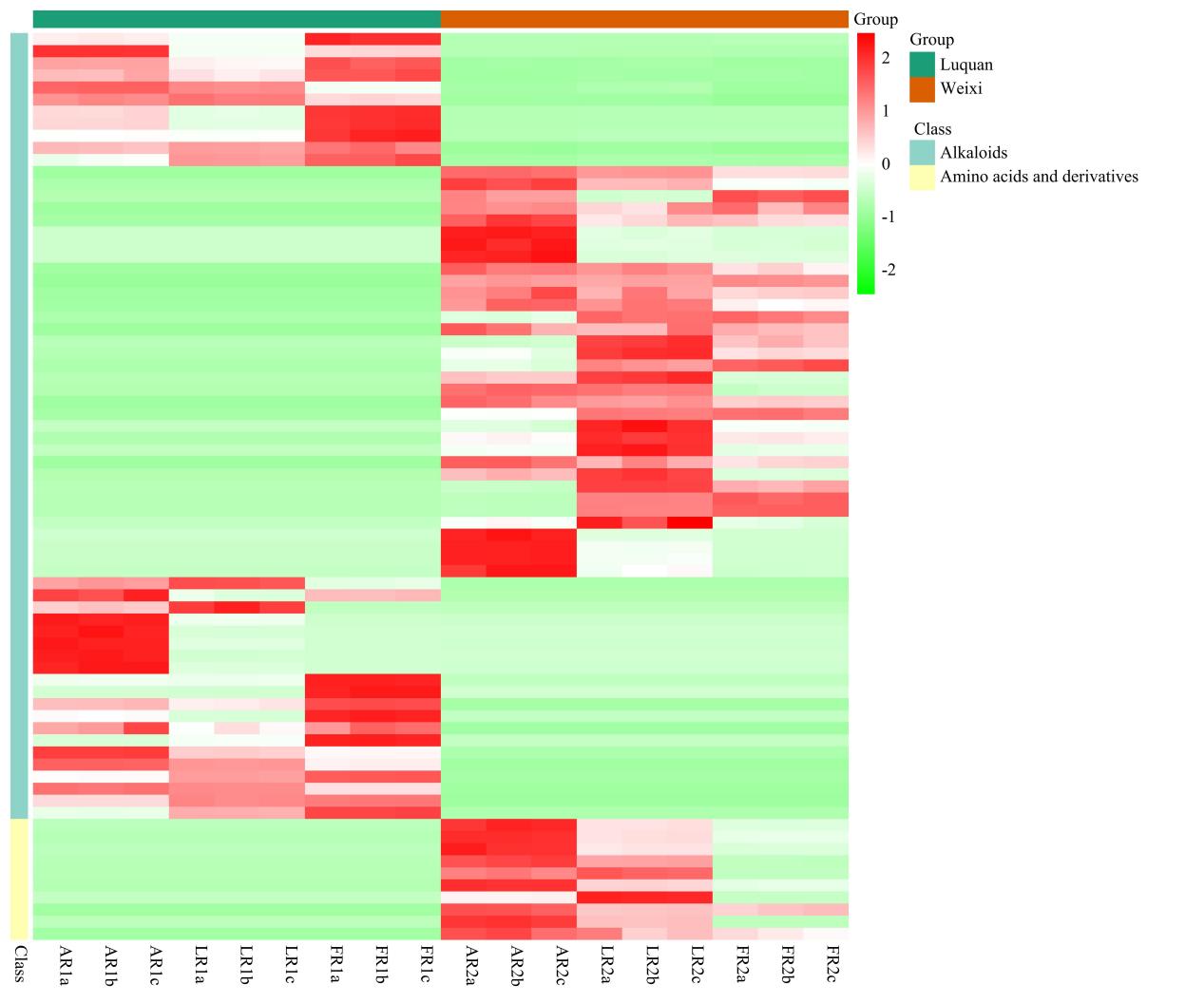


**Sup Fig. S2** The cluster heatmap of differential metabolites. The color scale indicated the abundance of metabolites.

**
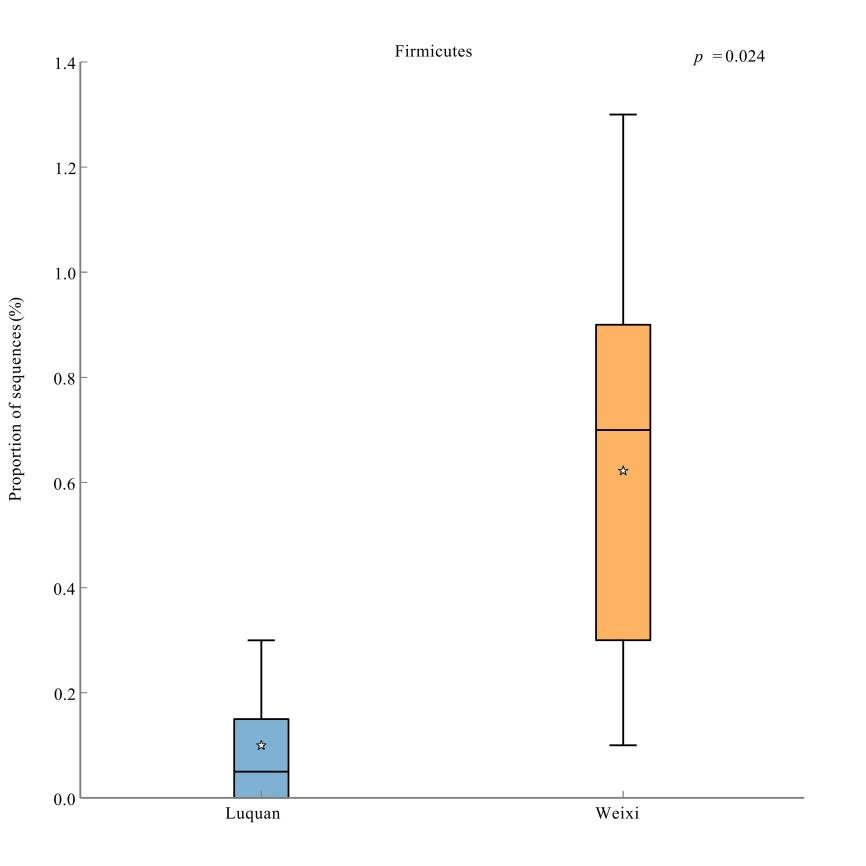
**

**Sup Fig. S3** The change of the relative abundance in Firmicutes. There was a significant difference in the relative abundance in Firmicutes of the samples between Luquan and Weixi (ANOVA, Tukey-HSD test).


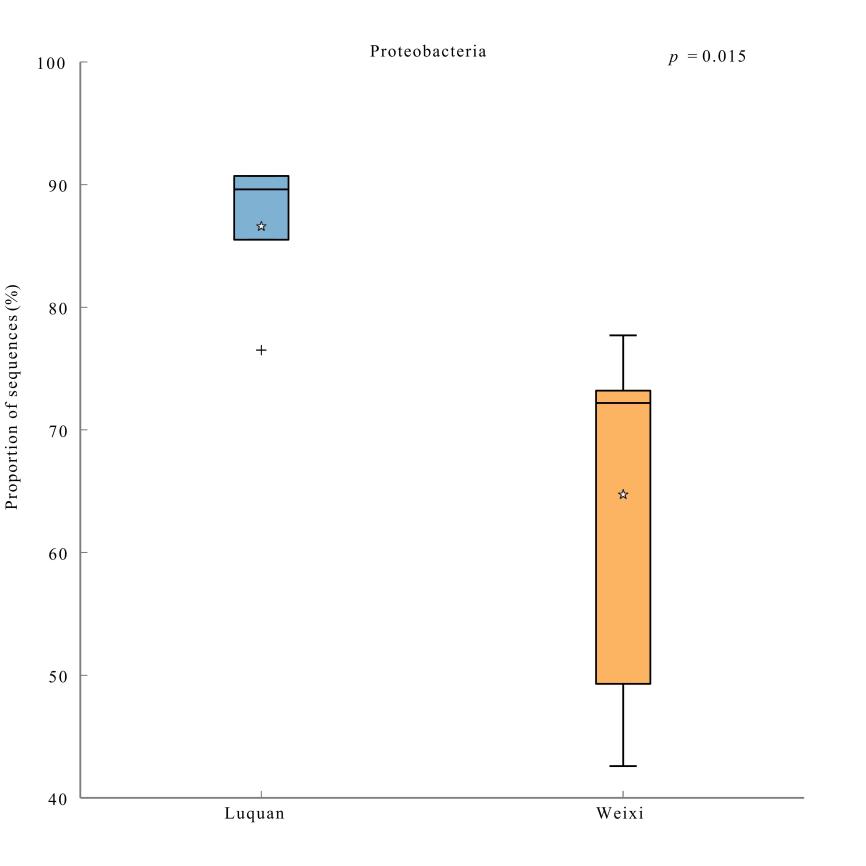


**Sup Fig. S4** The change of the relative abundance in Proteobacteria. There was a significant difference in the relative abundance in Proteobacteria of the samples between Luquan and Weixi (ANOVA, Tukey-HSD test).


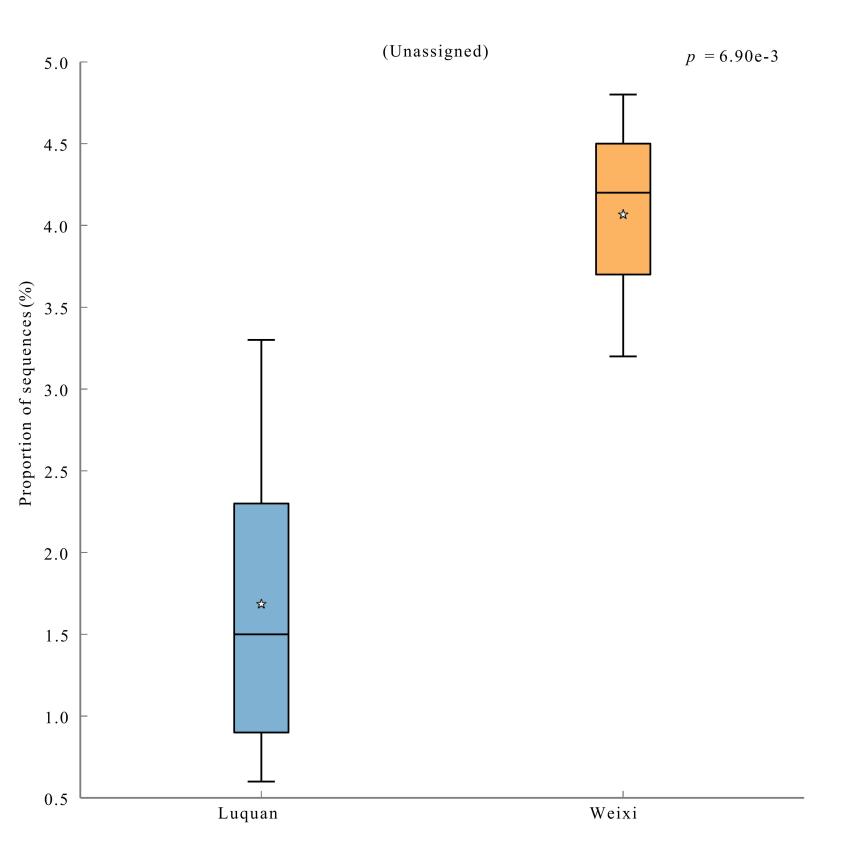


**Sup Fig. S5** The change of the relative abundance in unassigned fungi. There was a significant difference in the relative abundance in unassigned fungi of the samples between Luquan and Weixi (ANOVA, Tukey-HSD test).

**
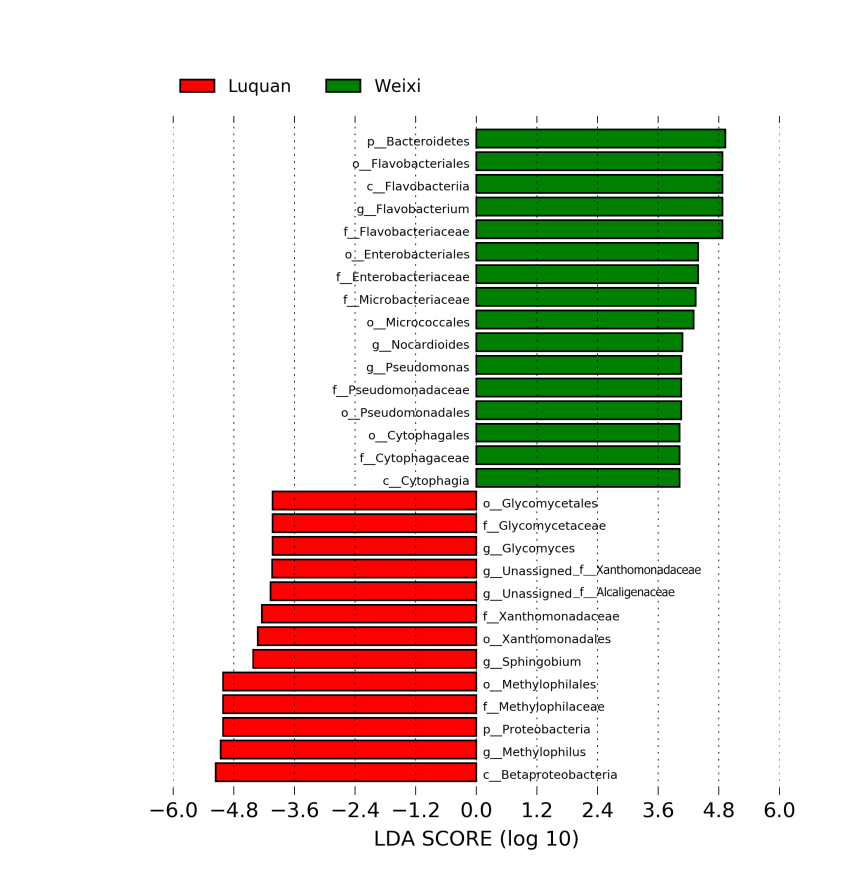
**

**Sup Fig. S6** The bacterial taxa with their LDA scores. Based on LEfSe results, the taxa were ranked according to their LDA scores.


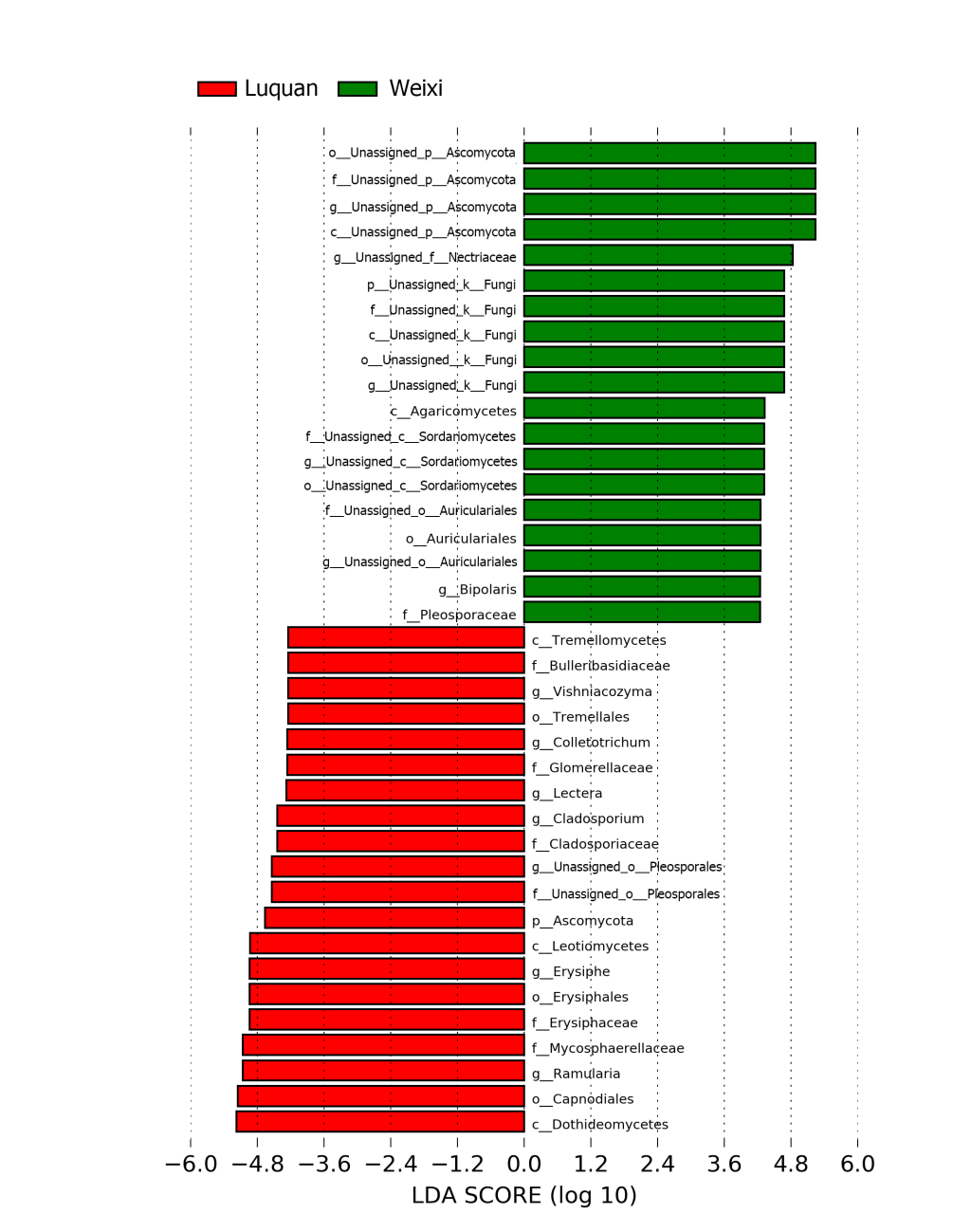


**Sup Fig. S7** The fungal taxa with their LDA scores. Based on LEfSe results, the taxa were ranked according to their LDA scores.

**Sup Fig. S8-S43** Total ions current (TIC) graphs of each sample. N stood for negative ion mode, P for positive ion mode.


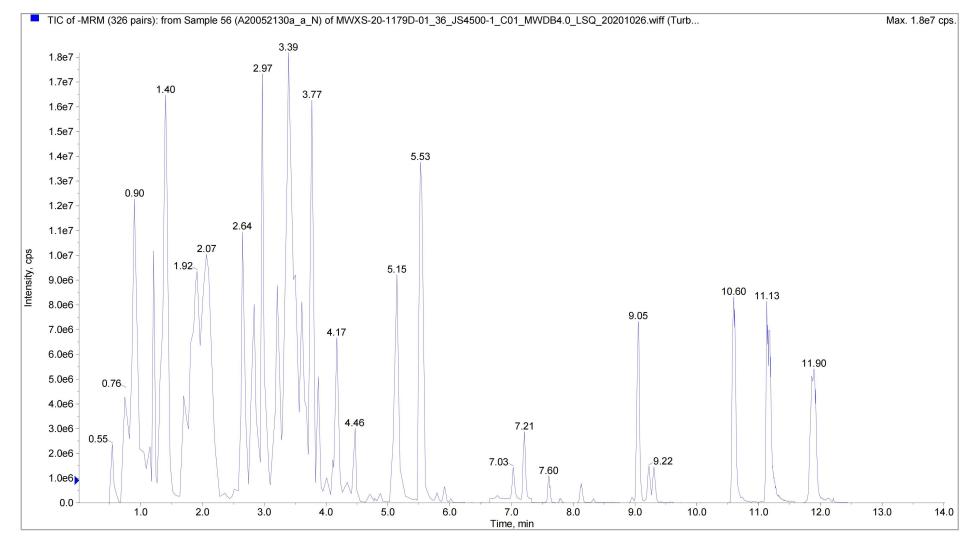


**Sup Fig. S8** TIC graphs of AR1a_N.

**
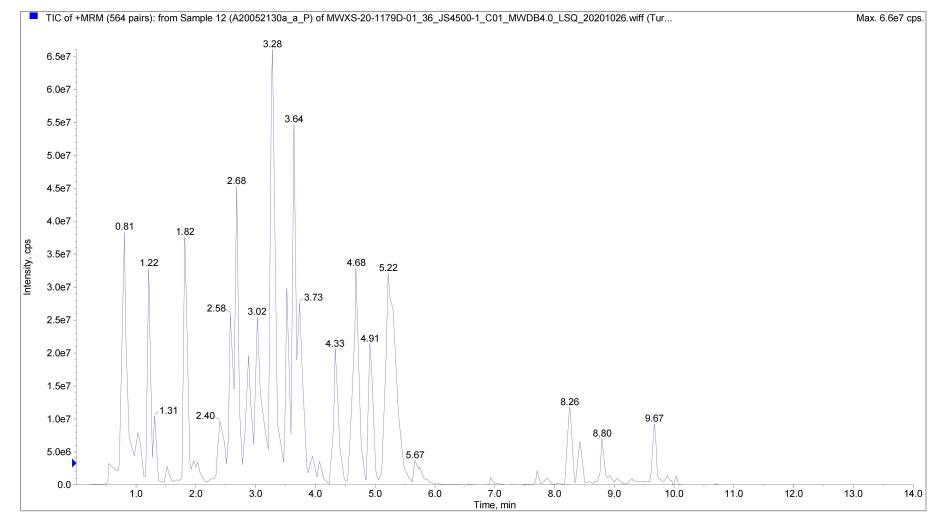
**

**Sup Fig. S9** TIC graphs of AR1a_P.

**
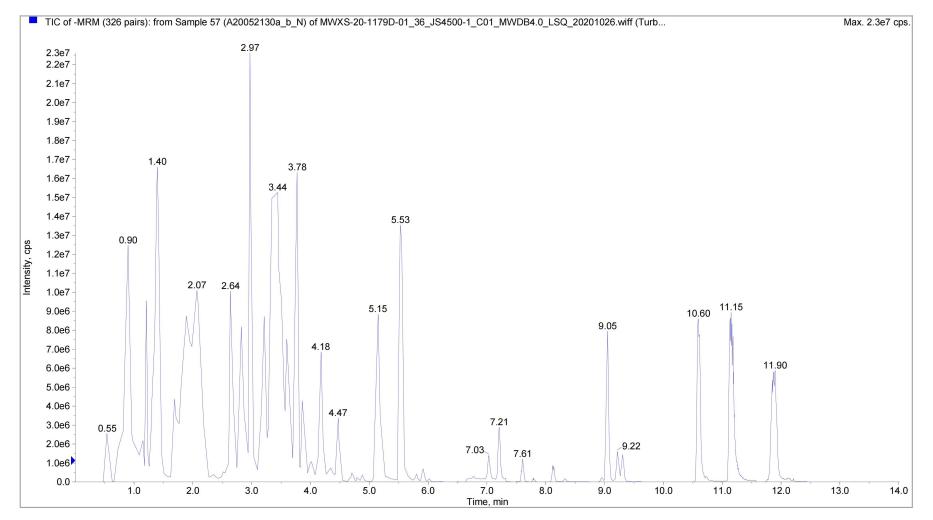
**

**Sup Fig. S10** TIC graphs of AR1b_N.

**
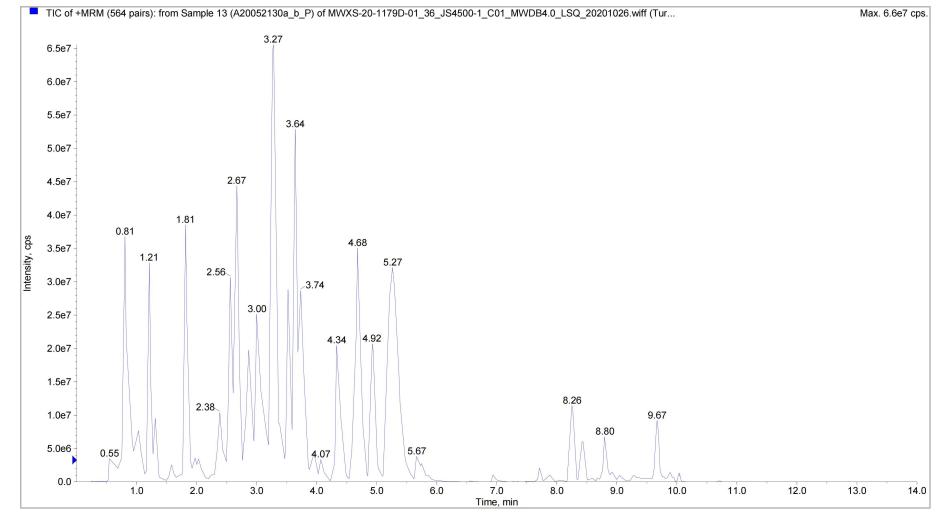
**

**Sup Fig. S11** TIC graphs of AR1b_P.

**
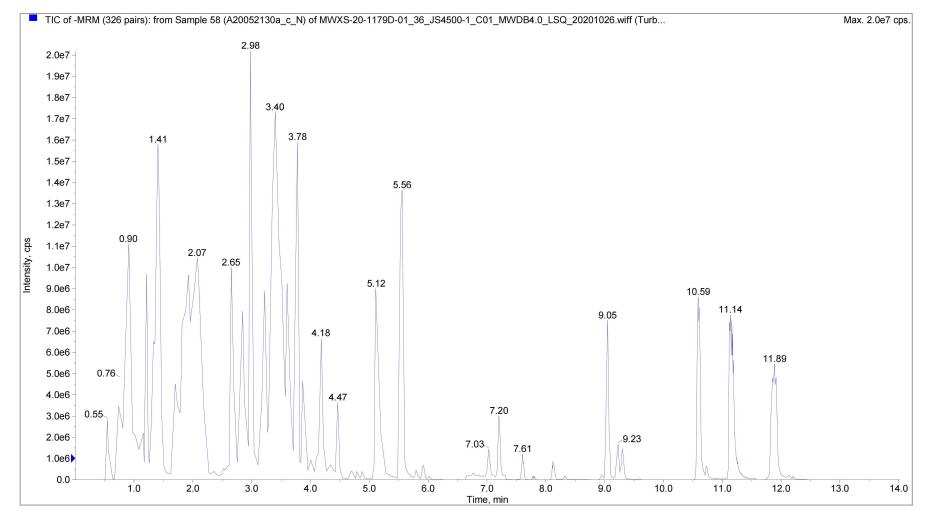
**

**Sup Fig. S12** TIC graphs of AR1c_N.

**
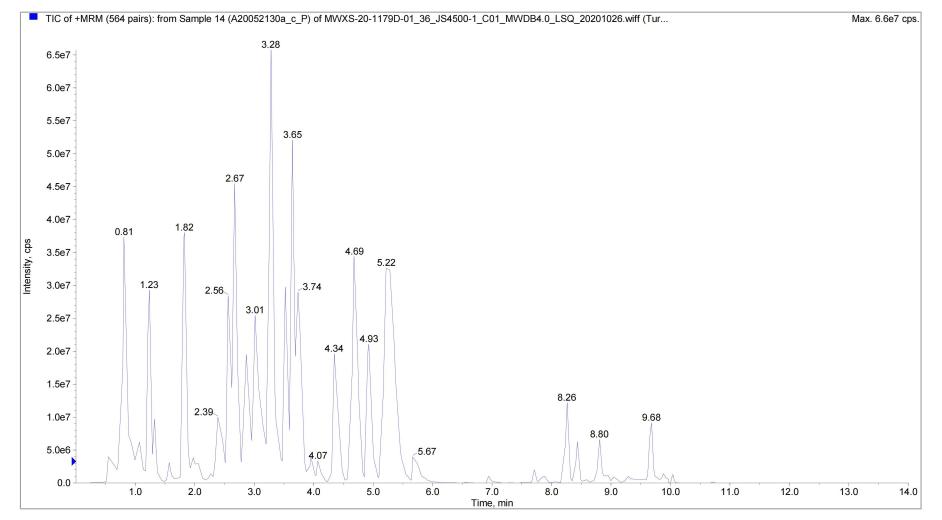
**

**Sup Fig. S13** TIC graphs of AR1c_P.

**
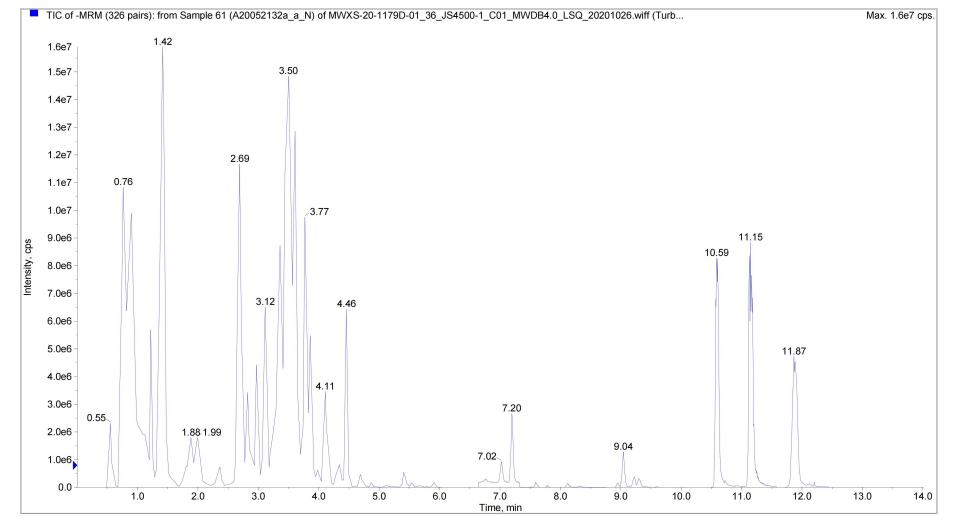
**

**Sup Fig. S14** TIC graphs of LR1a_N**.**

**
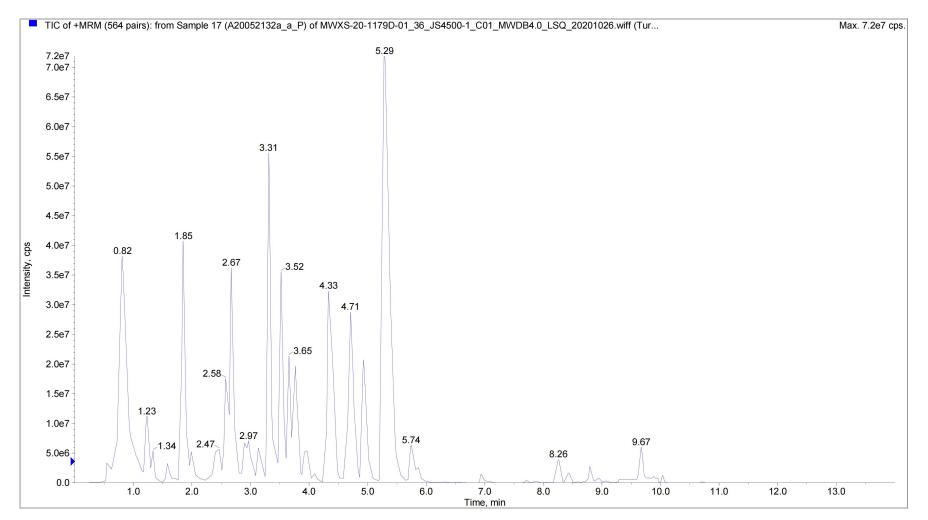
**

**Sup Fig. S15** TIC graphs of LR1a_P.

**
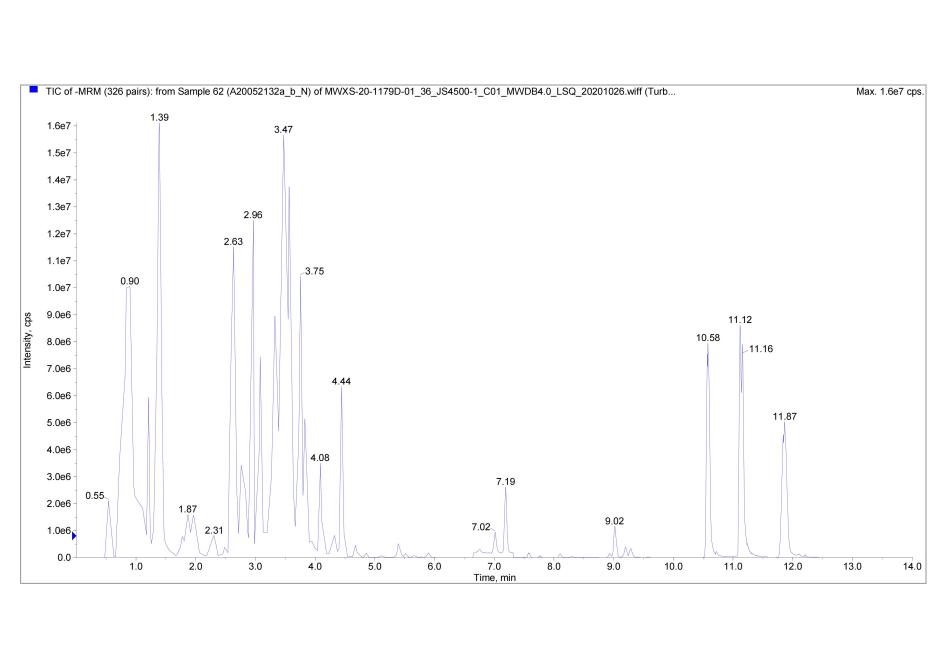
**

**Sup Fig. S16** TIC graphs of LR1b_N.


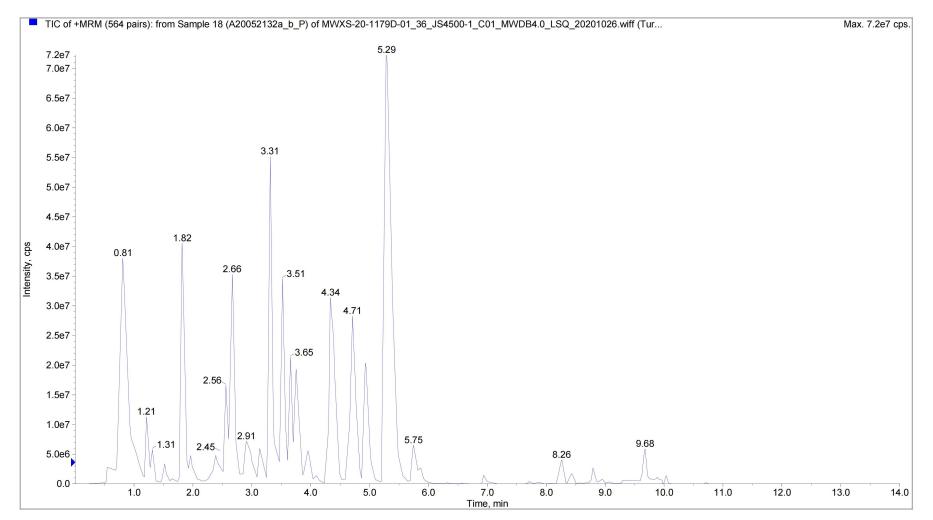


**Sup Fig. S17** TIC graphs of LR1b_P.


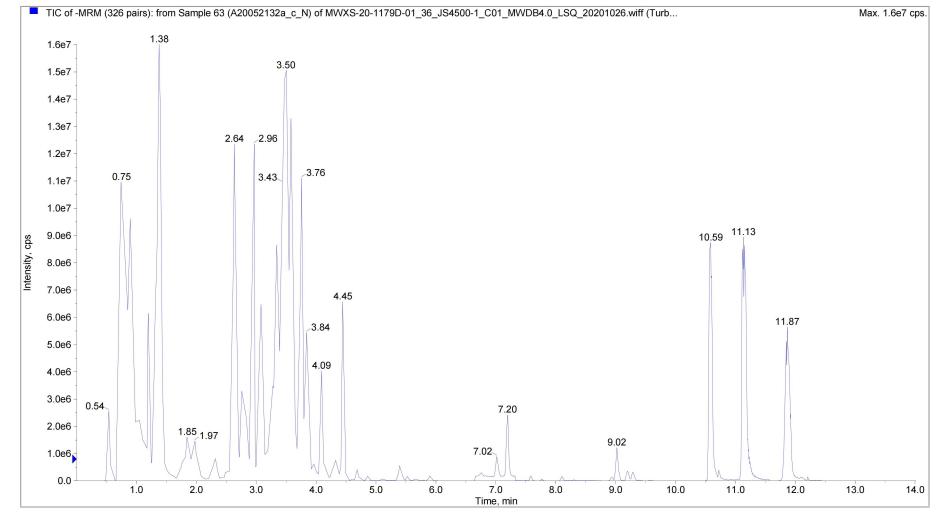


**Sup Fig. S18** TIC graphs of LR1c_N.


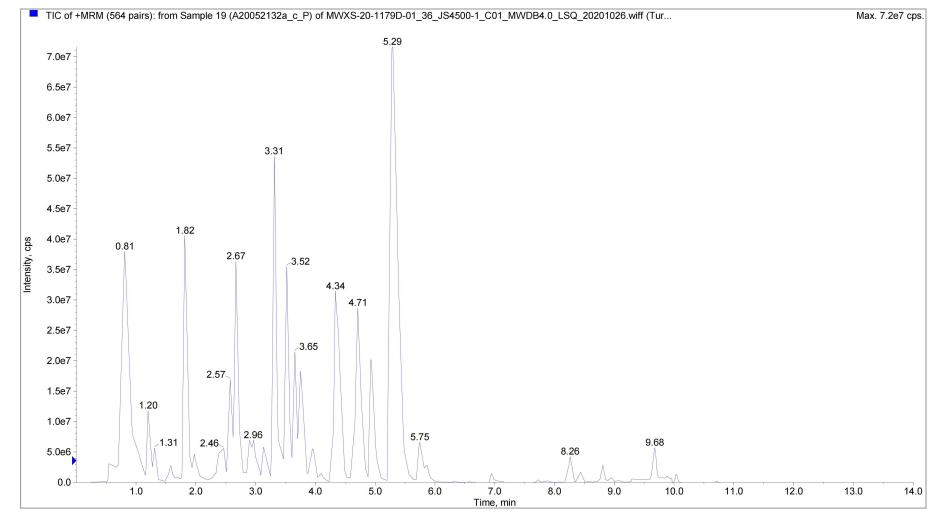


**Sup Fig. S19** TIC graphs of LR1c_P.


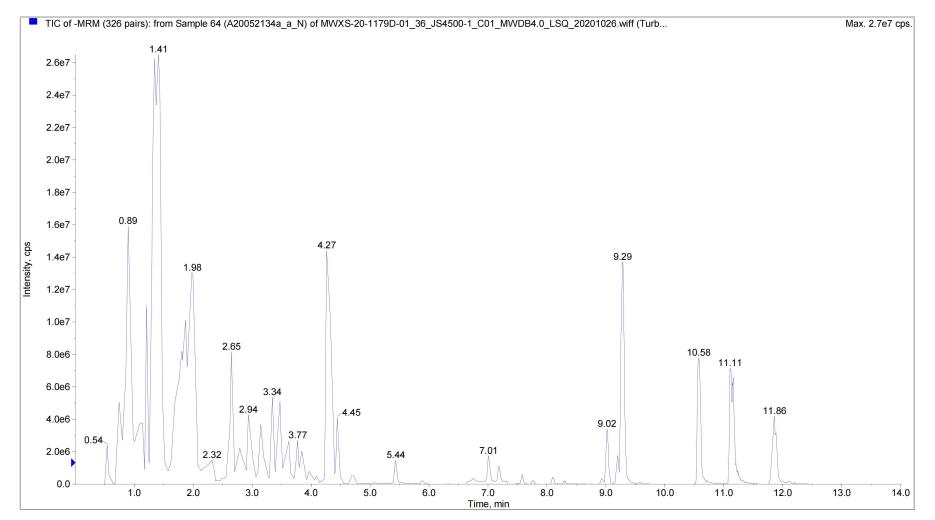


**Sup Fig. S20** TIC graphs of FR1a_N.


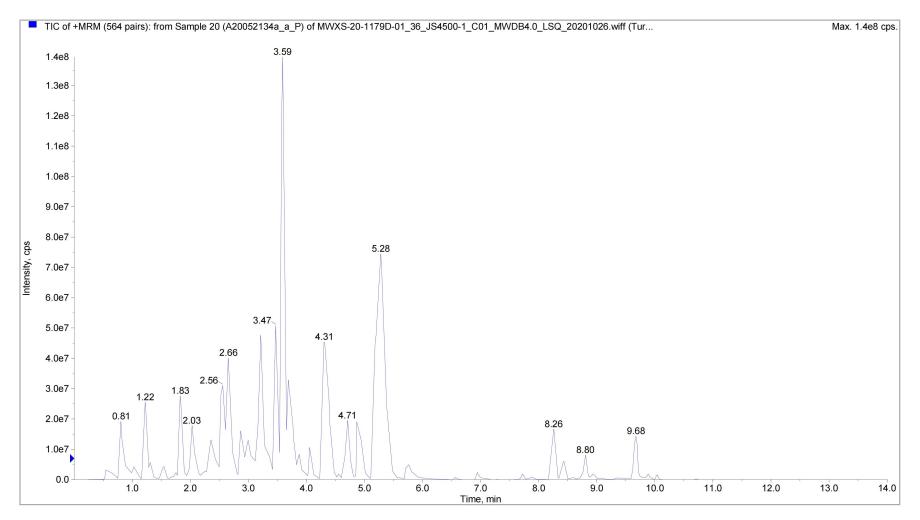


**Sup Fig. S21** TIC graphs of FR1a_P.


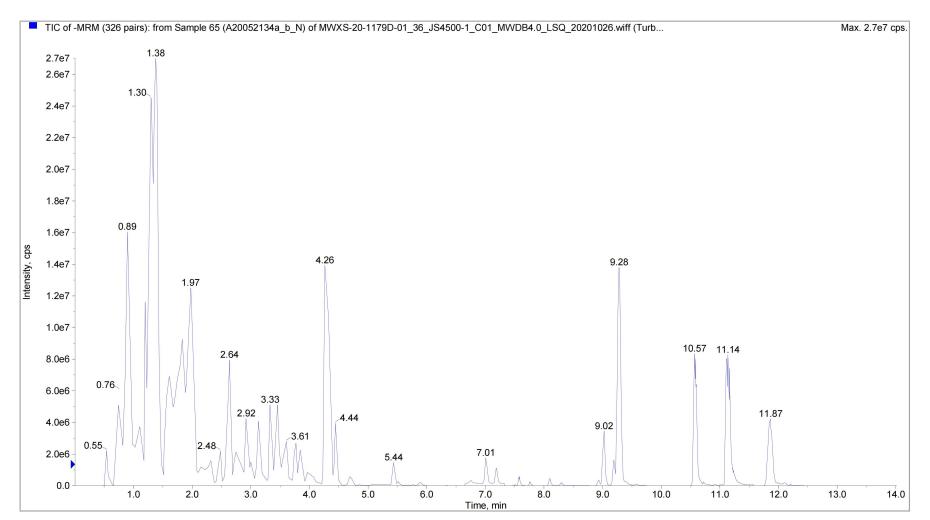


**Sup Fig. S22** TIC graphs of FR1b_N.


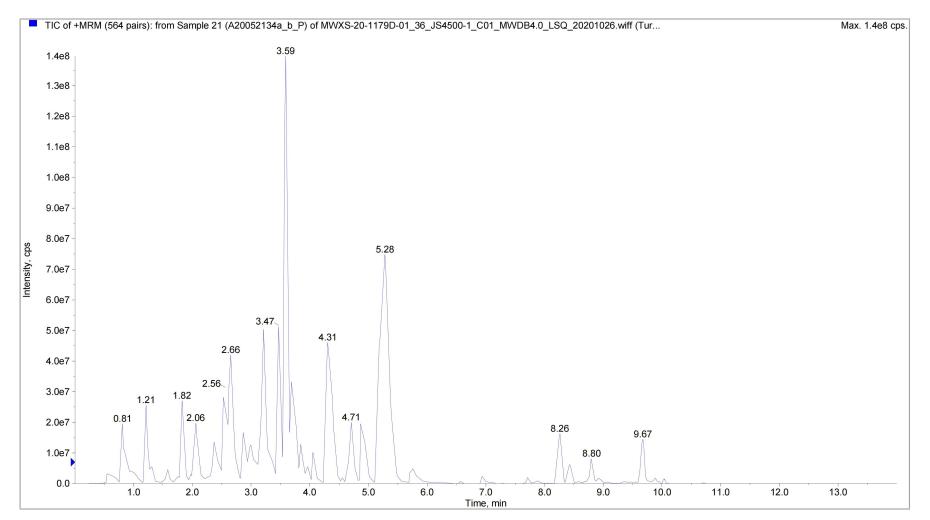


**Sup Fig. S23** TIC graphs of FR1b_P.


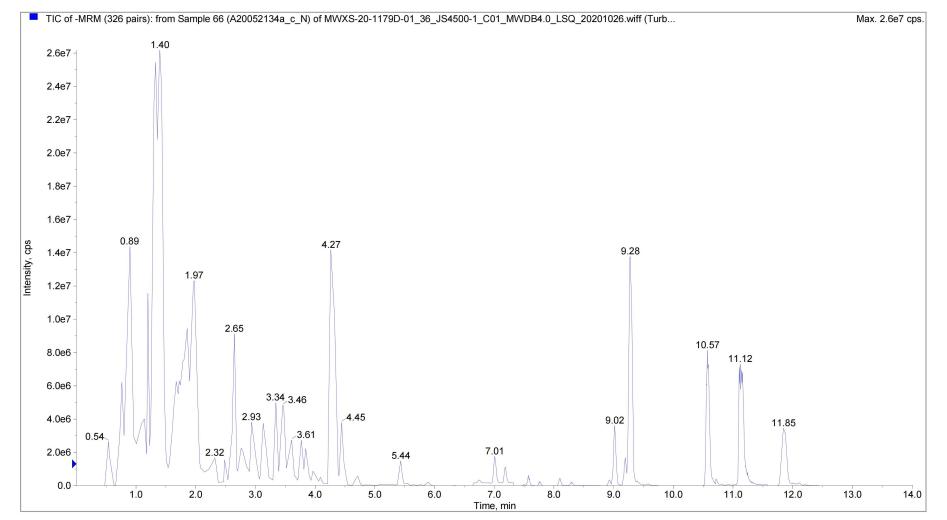


**Sup Fig. S24** TIC graphs of FR1c_N.


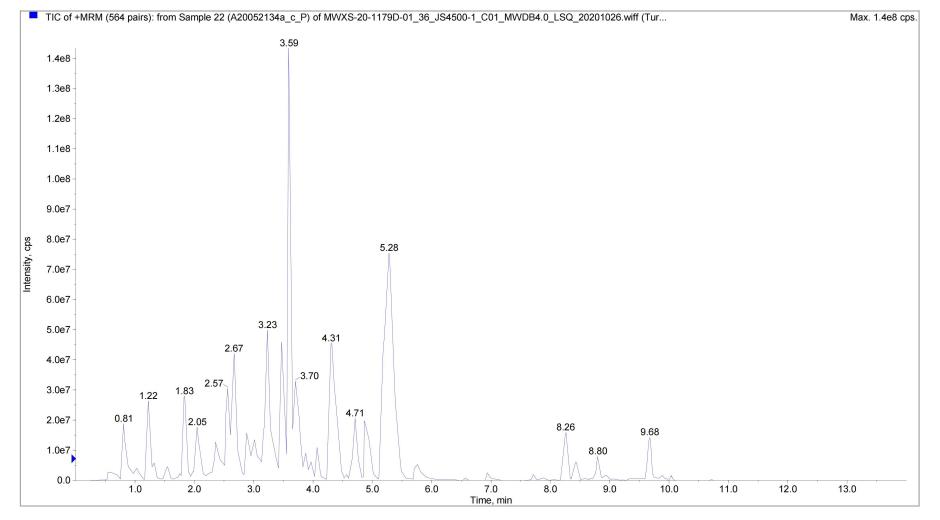


**Sup Fig. S25** TIC graphs of FR1c_P.

**
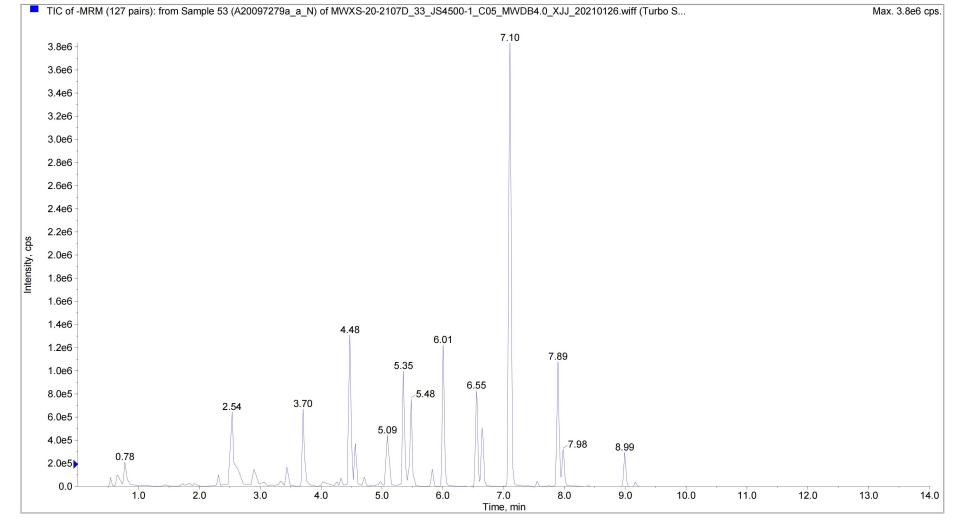
**

**Sup Fig. S26** TIC graphs of AR2a_N.


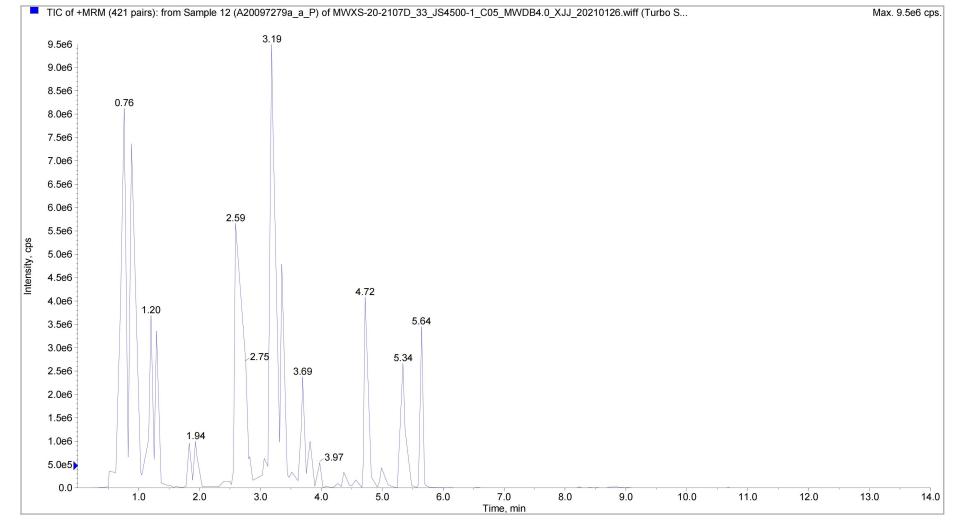


**Sup Fig. S27** TIC graphs of AR2a_P.


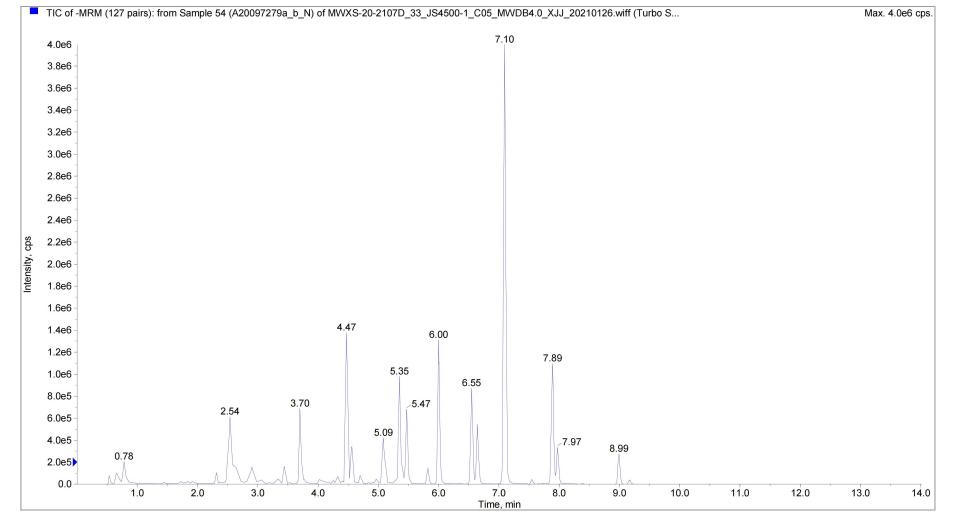


**Sup Fig. S28** TIC graphs of AR2b_N.


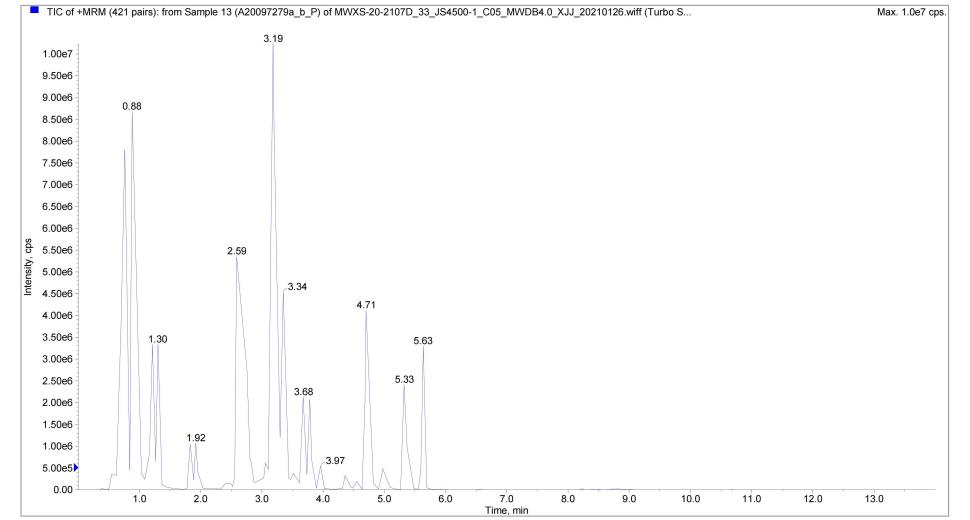


**Sup Fig. S29** TIC graphs of AR2b_P.


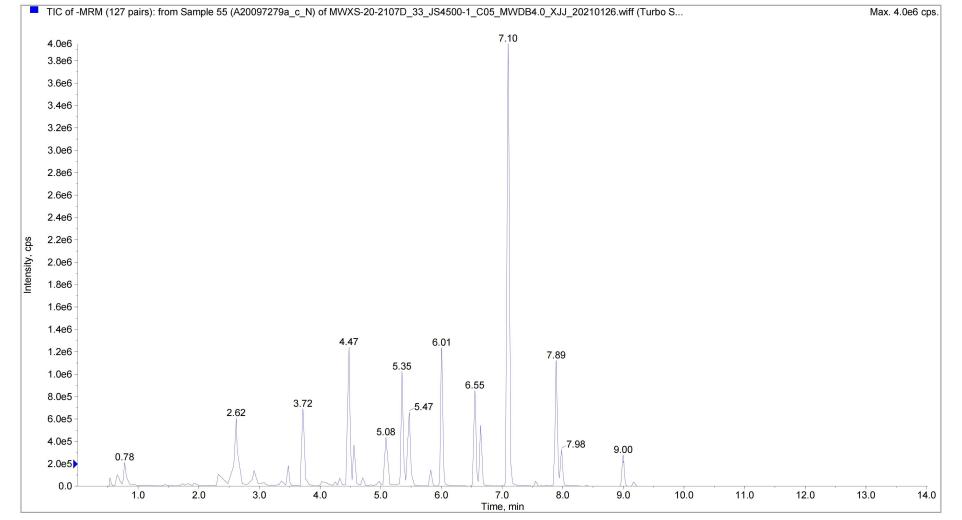


**Sup Fig. S29** TIC graphs of AR2c_N.


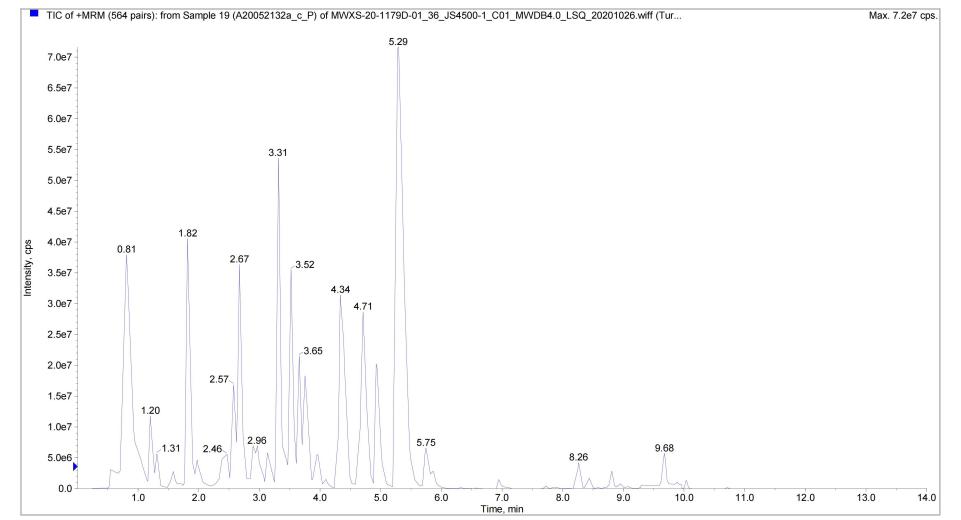


**Sup Fig. S31** TIC graphs of AR2c_P.

**
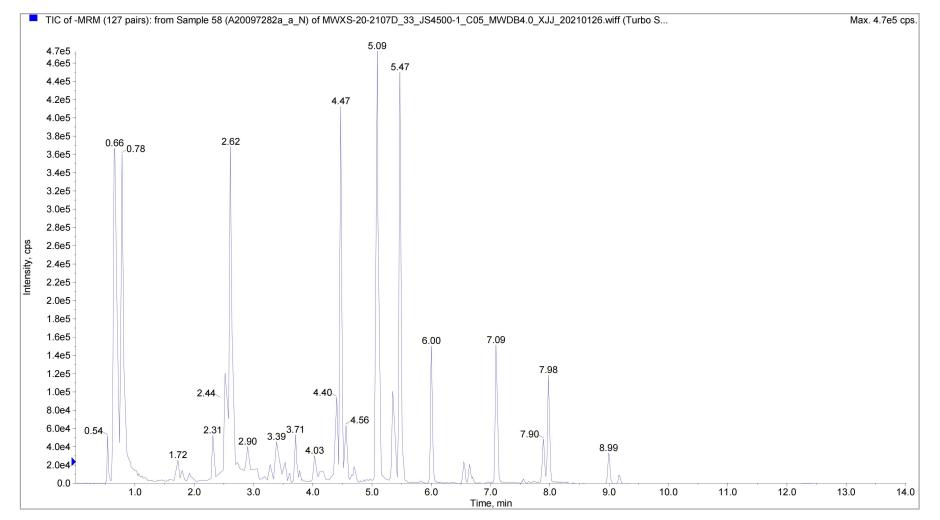
**

**Sup Fig. S32** TIC graphs of LR2a_N.


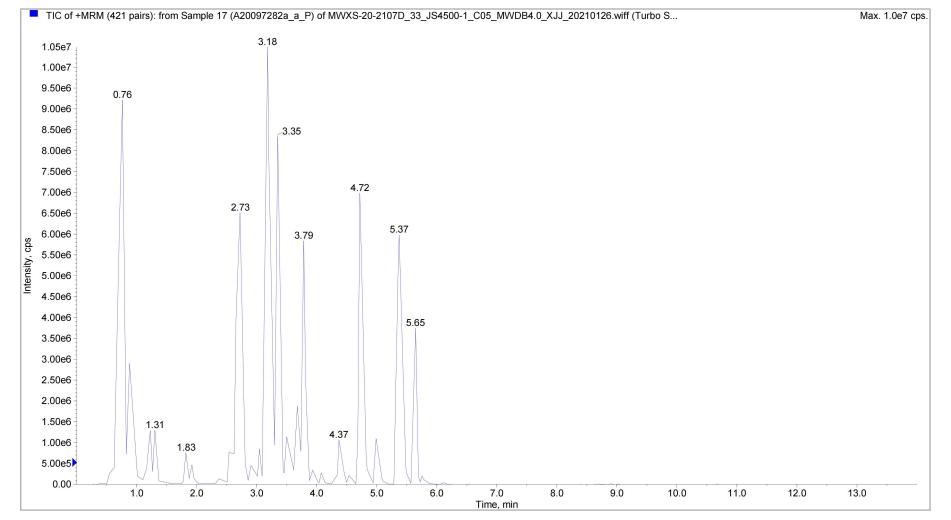


**Sup Fig. S33** TIC graphs of LR2a_P.


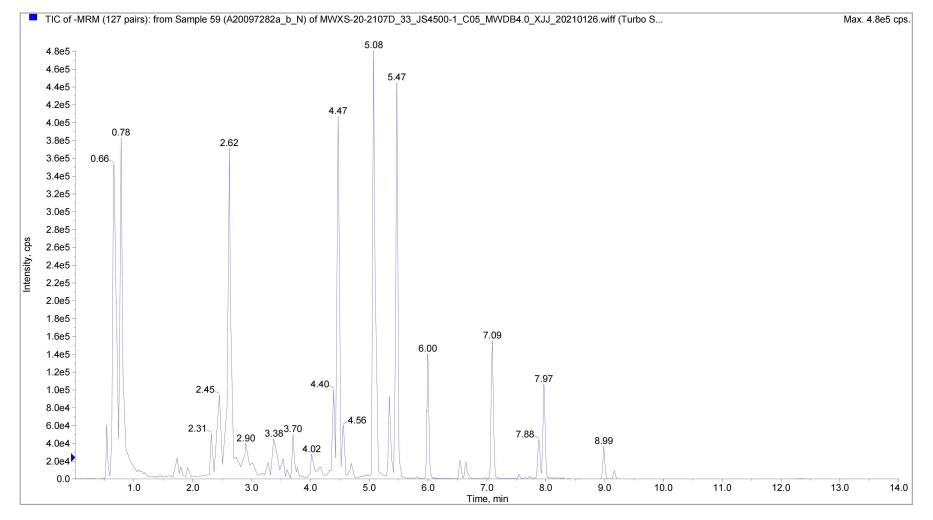


**Sup Fig. S34** TIC graphs of LR2b_N.


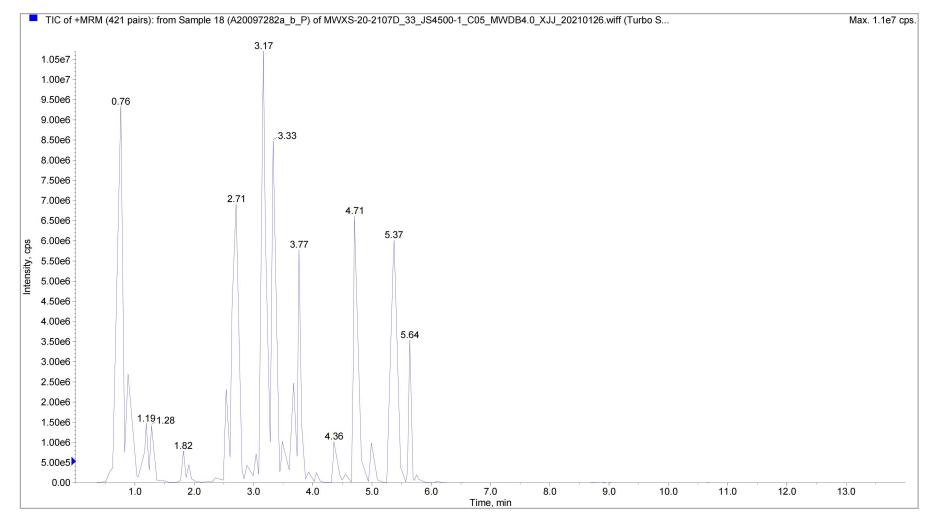


**Sup Fig. S35** TIC graphs of LR2b_P.


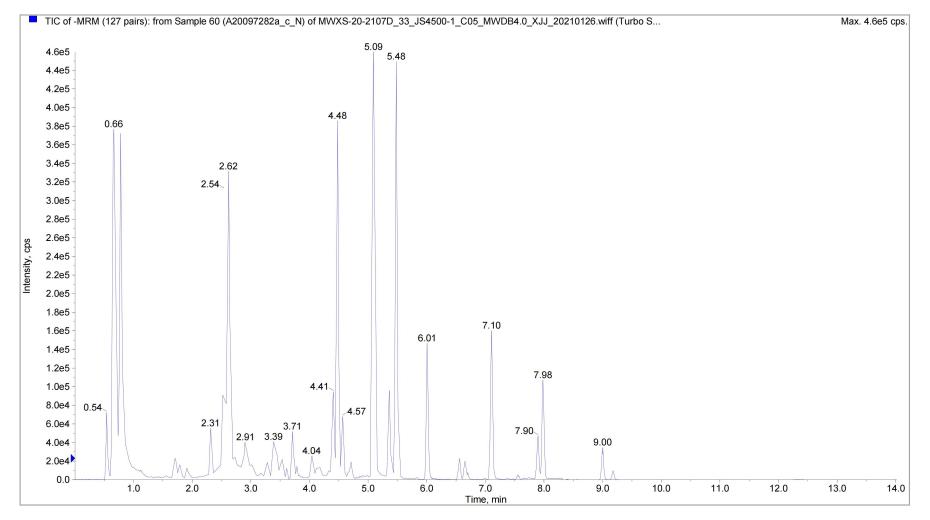


**Sup Fig. S36** TIC graphs of LR2c_N.


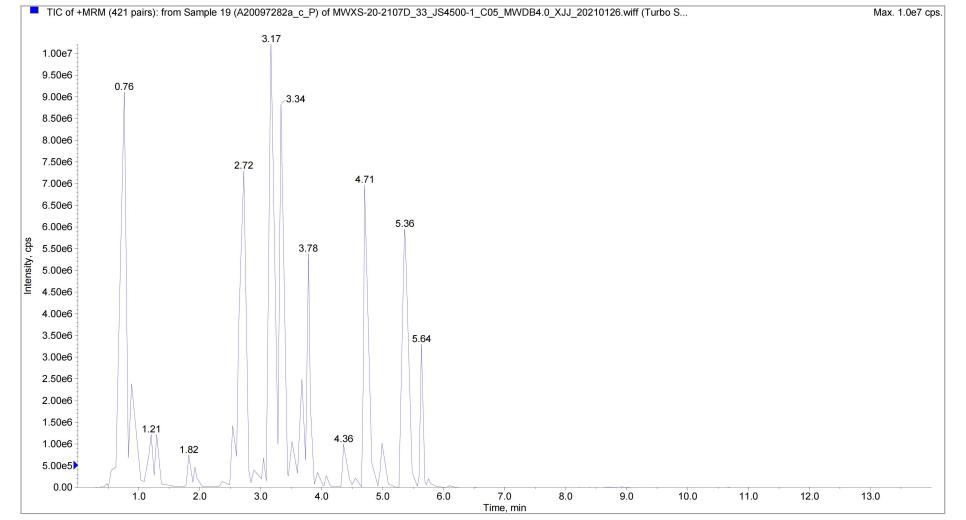


**Sup Fig. S37** TIC graphs of LR2c_P.


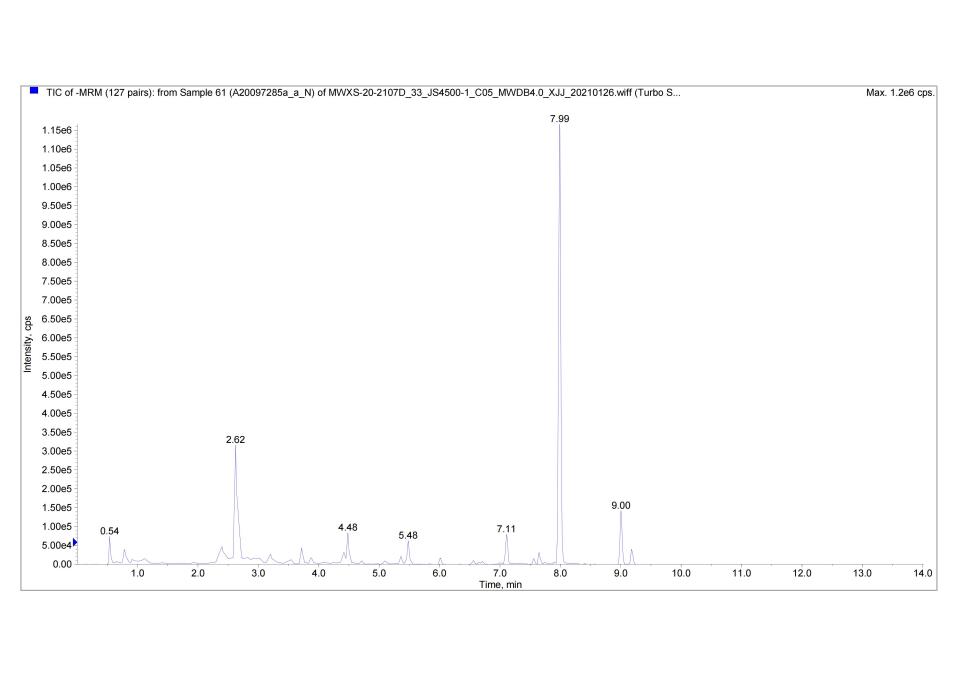


**Sup Fig. S38** TIC graphs of FR2a_N.


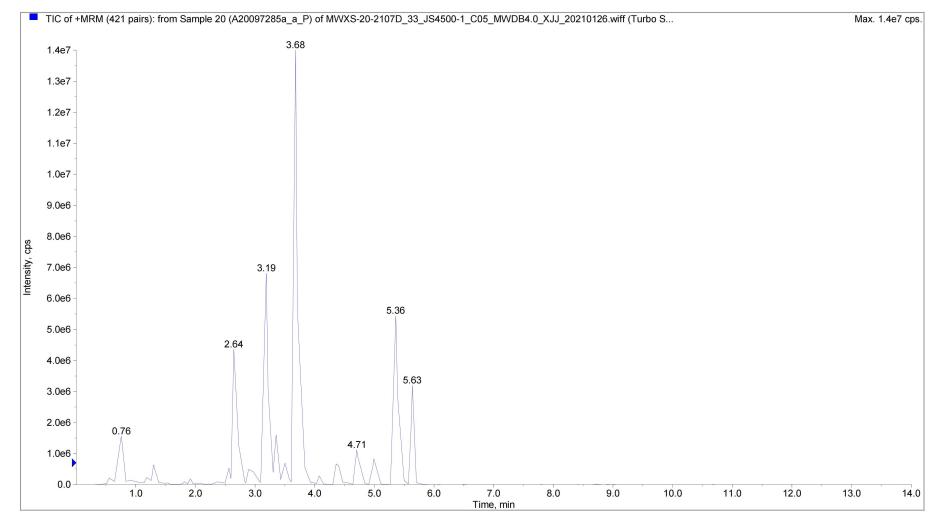


**Sup Fig. S39** TIC graphs of FR2a_P.


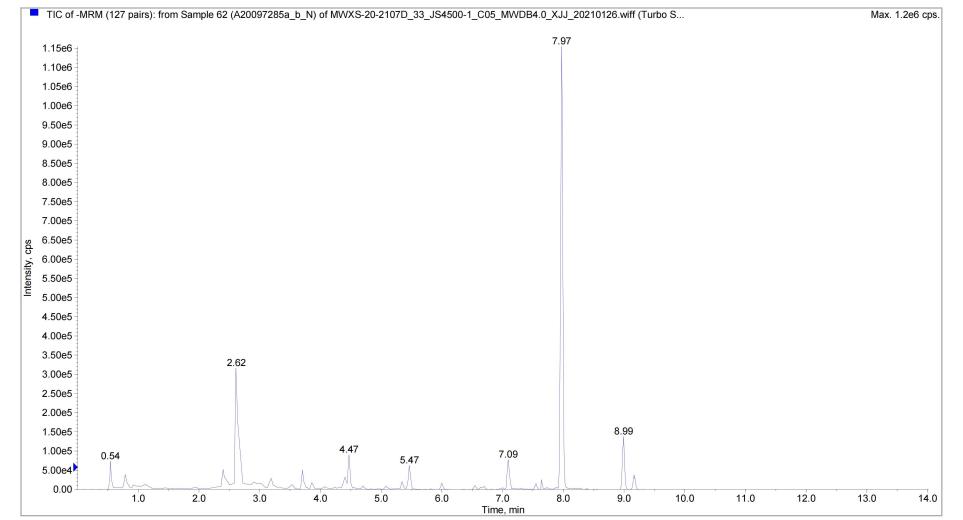


**Sup Fig. S40** TIC graphs of FR2b_N.


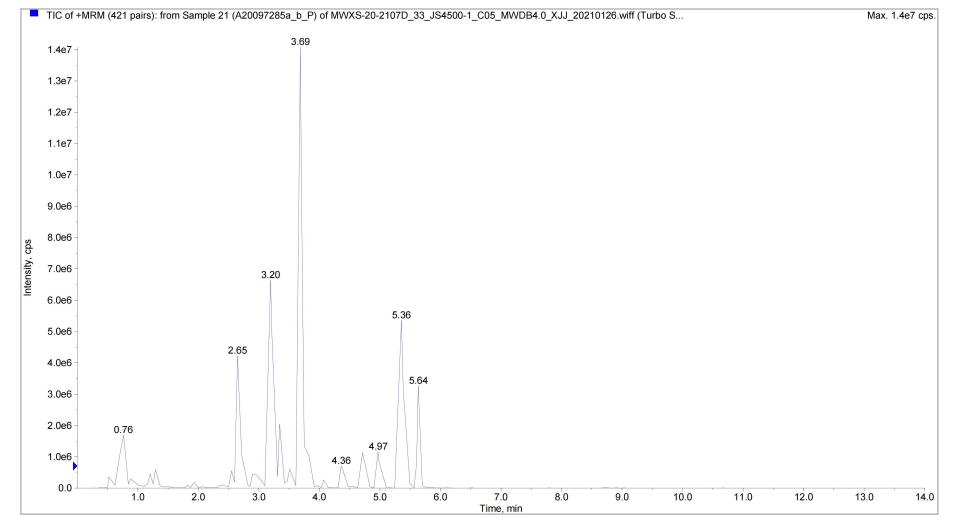


**Sup Fig. S41** TIC graphs of FR2b_P.


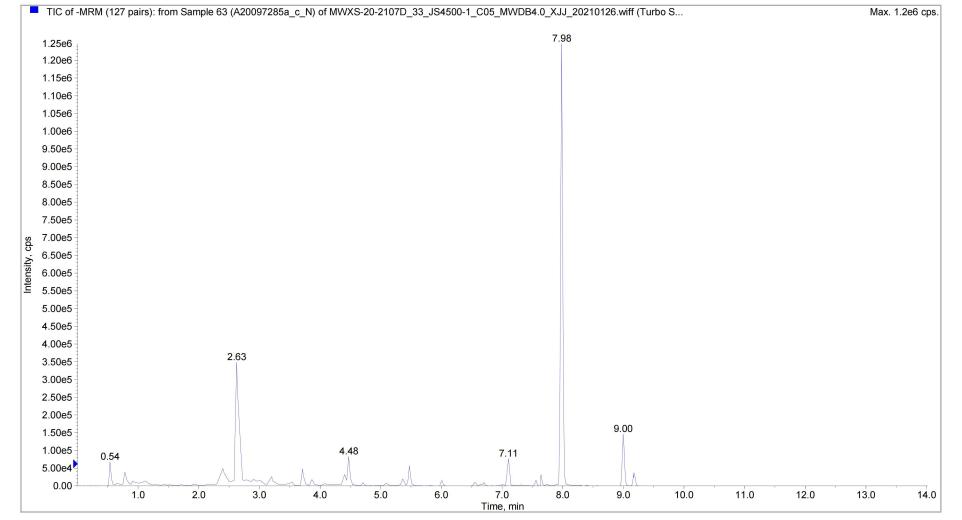


**Sup Fig. S42** TIC graphs of FR2c_N.

**
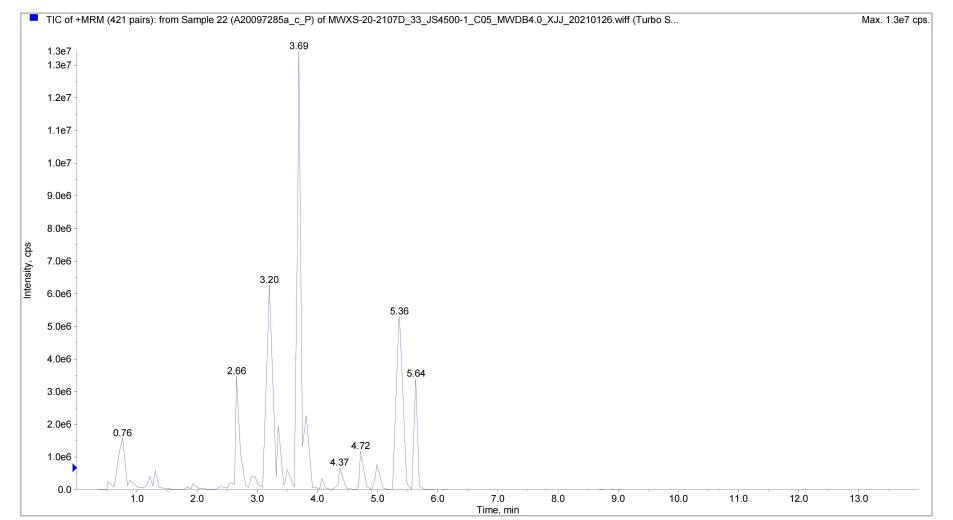
**

**Sup Fig. S43** TIC graphs of FR2c_P.
